# Supplementary material for: Systematic review and meta-analysis of the association between peripheral inflammatory cytokines and generalised anxiety disorder
Source: BMJ Open. 2019 Jul 19;9(7):e027925. doi: 10.1136/bmjopen-2018-027925 (PMC6661660; doi:10.1136/bmjopen-2018-027925)
Supplement: Supplementary data [file bmjopen-2018-027925supp001.pdf]

MEDLINE SEARCH STRATEGY:
(“inflammat*” OR “cytokine” OR “interferon” OR “IFN” OR “interleukin” OR “translocator protein” OR “TSPO” OR “tumour necrosis factor” OR “tumor necrosis factor” OR “TNF” OR “IL-1” OR “IL-2” OR “IL-4” OR “IL-7” OR “IL-6” OR IL-8 OR IL-10 OR migroglia OR t-cell OR lymphocyte OR “C-reactive protein” OR “C reactive protein” OR CRP OR “acute phase protein” OR “fibrinogen”) AND (“generalised anxiety disorder” OR “generalized anxiety disorder” OR “GAD” OR “worry”).
