# Supplementary material for: Systematic review and meta-analysis of the association between peripheral inflammatory cytokines and generalised anxiety disorder
Source: BMJ Open. 2019 Jul 19;9(7):e027925. doi: 10.1136/bmjopen-2018-027925 (PMC6661660; doi:10.1136/bmjopen-2018-027925)
Supplement: Author's manuscript [file bmjopen-2018-027925.draft_revisions.pdf]

BMJ Open

BMJ Open is committed to open peer review. As part of this commitment we make the peer review history of every article we publish publicly available.

When an article is published we post the peer reviewers' comments and the authors' responses online. We also post the versions of the paper that were used during peer review. These are the versions that the peer review comments apply to.

The versions of the paper that follow are the versions that were submitted during the peer review process. They are not the versions of record or the final published versions. They should not be cited or distributed as the published version of this manuscript.

BMJ Open is an open access journal and the full, final, typeset and author-corrected version of record of the manuscript is available on our site with no access controls, subscription charges or pay-per-view fees (http://bmjopen.bmj.com).

If you have any questions on BMJ Open's open peer review process please email info.bmjopen@bmj.com

BMJ Open

A systematic review and meta-analysis of the association between peripheral inflammatory cytokines and generalised anxiety disorder

Journal:	*BMJ Open*
Manuscript ID	bmjopen-2018-027925
Article Type:	Research
Date Submitted by the Author:	14-Nov-2018

Complete List of Authors:	Costello, Harry; University College London Division of Psychiatry,<br>Gould, Rebecca; University College London Division of Psychiatry<br>Abrol, Esha; University College London Division of Psychiatry<br>Howard, R; University College London Division of Psychiatry,

Keywords:	Anxiety disorders < PSYCHIATRY, Immunology < BASIC SCIENCES, PSYCHIATRY

SCHOLARONE™
Manuscripts

A systematic review and meta-analysis of the association between peripheral inflammatory cytokines and generalised anxiety disorder

H. Costello[1], R.L. Gould[1], E. Abrol, R.J. Howard[1]

[1] Division of Psychiatry, University College London, London W1T 7NF, United Kingdom

* Address correspondence to Dr. Harry Costello: h.costello@ucl.ac.uk

4603 words (excluding abstract: 274 words)

For peer review only

Abstract

Aim: To systematically review and meta-analyse studies measuring peripheral cytokine levels in people with generalised anxiety disorder (GAD) compared to controls.

Methods: We searched MEDLINE (1950–), EMBASE (1947-), PsycINFO (1872–) and Web of Science (1945–) databases up until January 2018. Studies that met inclusion criteria were assessed for quality and data were extracted. Meta-analysis using a random-effects model was conducted for individual cytokines where sufficient data from three or more studies were available.

Results: 14/1718 identified studies met inclusion criteria, comprising 1188 patients with GAD and 10,623 controls. In total 16 different cytokines were evaluated. Significantly raised levels of CRP, IFN-γ and TNF-α were reported in GAD patients compared to controls in two or more studies. Ten further pro-inflammatory cytokines were reported to be significantly raised in GAD in at least one study. However, 5/14 studies found no difference in levels of at least one cytokine. Only CRP studies reported sufficient data for meta-analysis. A significantly higher level of CRP was found in people with GAD compared to controls, with a small effect size (Cohen's d = 0.38, 0.06-0.69), comparable to that reported in schizophrenia. However, heterogeneity was high (I^2= 75%), in keeping with meta-analyses of inflammatory markers in other psychiatric conditions and reflecting potential differences in mediators of inflammation including medication use, co-morbid depression and cytokine sampling methodology.

Conclusion: There is preliminary evidence to suggest an inflammatory response in GAD, but it remains unclear whether inflammatory cytokines play a role in aetiology. GAD remains a poorly studied area of neuroinflammation compared to other mental disorders and further longitudinal studies confirming and characterising the role and profile of inflammation are required.

Strengths and limitations of this study

- This is a comprehensive systematic review, and the first meta-analysis, of peripheral cytokine levels in people with generalised anxiety disorder (GAD).
- Our review examined the cross-sectional and longitudinal associations between inflammatory biomarkers and GAD.
- Relatively few studies, of variable quality, were identified and only one study examined the longitudinal association between inflammation and GAD.
- Only studies measuring C-reactive protein reported sufficient data for meta-analysis.
- We were unable to analyse publication bias due to the paucity of studies included in meta-analysis.

Introduction

There is growing evidence for immune mediated pathogenic mechanisms in several psychiatric disorders with discrete profiles of inflammatory mechanisms[1]. Epidemiological evidence has shown an increased risk of mood disorders and psychosis in people with a history of severe infection or autoimmune conditions[2,3]. This has been supported by genome wide association studies implicating multiple immune signalling pathways[4], and altered

For peer review only

profiles of pro-inflammatory cytokines and acute phase reactants in schizophrenia[5], depression[6], obsessive compulsive disorder (OCD)[7] and bipolar disorder[8]. However, the relationship between inflammation and mental illness remains poorly understood and controversial, with a number of proposed potential neuropathological mechanisms[9,10], including changes in microglial function[1], glutamatergic excitotoxicity[11], synaptic plasticity[12] and reduced hippocampal neurogenesis[13].

Despite increasing interest in the role of inflammation in mental illness, relatively little research has focused on potential associations with anxiety disorders[14]. These are common, with an estimated lifetime prevalence of 7.3% to 28.8%, are associated with substantial functional impairment and are estimated to cost between 42-47 billion dollars to the U.S economy each year[15,16]. However, only 60% of patients are thought to respond to pharmacological and psychological treatments and understanding of the underlying pathophysiological mechanisms of anxiety disorders remains poor[17].

Generalised anxiety disorder (GAD) is the most common anxiety disorder, with a degree of associated disability equivalent to that of major depressive disorder (MDD)[18]. Despite psychopharmacological[19] and psychological[20] treatments showing effectiveness in GAD, 42% of people living with GAD experience ongoing symptoms after 12 years and half of remitted patients experience recurrence[18]. GAD is more prevalent in those with inflammatory conditions such as rheumatoid arthritis (RA)[21,22], with case series studies suggesting symptoms are less common with immune modulating treatment targeting specific inflammatory cytokines[23]. The chronic clinical course and relatively high probability of recurrence in GAD, in addition to preliminary evidence of an inflammatory component in other anxiety disorders[7,24], suggest that inflammation could be an important neurobiological mechanism in the aetiology of this disorder.

To date, two previous reviews of inflammatory biomarkers in GAD have been conducted. Of these, however, one was a narrative review [25] and the other was restricted to literature published within the last decade [14], and with a focus on all anxiety disorders. Both reviews reported that there was preliminary evidence for inflammatory changes in GAD. However, only three studies were identified by the systematic review reporting cytokine changes in GAD and no meta-analysis was performed. No study to date has conducted a comprehensive systematic review and meta-analysis of all current literature focusing on GAD or commented on the longitudinal association between inflammation and GAD.

We aimed to systematically review the cross-sectional and longitudinal associations between inflammatory biomarkers and GAD, and perform the first meta-analysis of inflammatory biomarkers in GAD.

Method

We conducted a systematic review of studies that had included people with GAD who had undergone peripheral cytokines measurement and a between group meta-analysis of cytokine

For peer review only

levels in people with GAD compared to controls. We conducted the study according to Preferred Reporting Items for Systematic Reviews and Meta-analyses (PRISMA) guidelines[26].

We searched MEDLINE (1950–), EMBASE (1947-), PsycINFO (1872–) and Web of Science (1945–) databases up until January 2018. Reference lists of eligible studies were then searched for further ones that met eligibility criteria.

Our search terms were: (inflammat* or cytokine or interferon or IFN or interleukin or "translocator protein" or TSPO or "tumour necrosis factor" or "tumor necrosis factor" or TNF or IL-1 or IL-2 or IL-4 or IL-7 or IL-6 or IL-8 or IL-10 or migroglia or t-cell or lymphocyte or "C-reactive protein" or "C reactive protein" or CRP or "acute phase protein" or "fibrinogen") and ("generalised anxiety disorder" or "generalized anxiety disorder" or GAD or worry).

We included primary, quantitative research studies (including unpublished theses and dissertations), written in any language, that included people with a diagnosis of GAD assessed using standardised clinical interview (e.g. Structured Clinical Interview for DSM[27]) or standardised psychometric instruments. Studies reported cross-sectional or longitudinal data in clinical or community populations. Cross-sectional studies measured inflammatory biomarker concentrations in anxious people versus non-anxious healthy controls, while longitudinal studies measured inflammatory biomarker concentrations at baseline and anxiety scores at follow-up. Inflammatory markers were measured in the unstimulated state (no antigen induced stimulation of cytokine production) and sampled from peripheral blood, CSF, or saliva at any time of day. Exclusion criteria included studies with less than 5 participants, studies in animals and studies where subjects were participants in the treatment arms of clinical trials.

Data extraction and quality assessment

Data were extracted and quality assessed for all studies that met eligibility criteria by two independent raters (HC, EA) with disagreements settled by consensus and discussion. For each cytokine, we extracted the means, variance estimates or 95% confidence intervals (CIs) and sample size for GAD and control groups. We also extracted demographic data (e.g. age, sex) and clinical data (e.g. medication use, co-morbid depression, severity) where available. Authors were contacted for further information, where necessary.

Risk of bias and study quality were evaluated using the Newcastle–Ottawa Quality Assessment Scale[28]. Other potential confounding factors (including assay type and sensitivity, inflammatory marker analysis and recruitment methods) were also examined to allow more detailed bias and quality analysis of studies.

For peer review only

Strategy for data synthesis

Separate meta-analyses were performed for individual biomarkers in GAD versus controls if sufficient data were available from a minimum of three studies. Due to different measurement methods and anticipated high heterogeneity, we estimated a standardised mean difference (SMD) for each inflammatory marker, and used a random effects model for meta-analysis, conducted using Revman 5. Heterogeneity across studies was quantified with the I^2 statistic, with a value of 25% typically regarded as low, 50% as medium, and 75% as high[29]. If studies were longitudinal or trials of interventions with multiple data collection points, we examined baseline data only to avoid skewed meta-analysis from inclusion of more than one effect size from the same study.

Results

Systematic review

We identified 1718 papers, excluded 1598 of these by titles and abstracts, and retrieved the remaining 120 papers, of which 14 met eligibility criteria and were included in the final systematic review (see Figure 1)[30–43]. The primary reasons for rejection were that no diagnosis of GAD was recorded or no inflammatory marker was measured.

Characteristics of the 14 included studies are shown in Tables 1 and 2. Studies comprised a total of 1188 people with a diagnosis of GAD and 10,623 controls, with a further 116 participants from a study that did not report GAD and control group sizes.

In total, 16 different cytokines were evaluated (see Table 2) C-reactive protein (CRP) (9/14 studies, 64.2%), tumour necrosis factor-α (TNF-α) (6/14 studies, 42.9%), IL-6 (5/14 studies, 35.7%) and interferon-γ (IFN-γ) (3/14 studies, 21.4%) were most commonly studied. All other cytokines were only analysed in two or less studies.

Twelve studies (85.7%) reported the assay method used, all of which were versions of an enzyme-linked immunosorbent assay (ELISA) or enzyme immunoassay (EIA). However, only 7 studies (50%) reported assay sensitivity. All but one study used blood component samples to assess inflammatory marker levels, with the most common sample type being serum (N=6, 42.9%) and plasma (N=4, 28.6%).

Risk of bias and quality in individual studies

All included studies had adequate case definition, with participants meeting diagnostic criteria for GAD according to DSM or ICD, with 12 studies (85.7%) using a structured clinical interview for assessment (see Table 1).

Most (71.4%) studies included people aged 18-65, but two studies (14.3%) only included participants over the age of 50 and a further two studies (14.3%) used adolescent participant cohorts. The majority (78.6%) of studies accounted for age and sex differences in their analyses. Only 8/14 studies (57.1%) recorded participants' body mass index (BMI) which is

For peer review only

known to correlate with inflammation, and only half of these accounted for BMI differences in analysis of group differences[44] (see Table 2).

Use of psychotropic medication and presence of comorbid major depressive disorder are important moderators of inflammation in other psychiatric disorders[24]. Six studies (42.8%) excluded patients who used psychiatric or other immune-modulating medication, though only two studies (4.3%) reported medication use. The majority of studies (64.2%) either excluded patients with co-morbid MDD or adjusted for this in analyses.

Concurrent physical illness is clearly an important determinant of inflammatory cytokine levels and this was accounted for by the majority of included studies by either excluding participants with co-morbidities (5 studies, 35.7%) or adjusting for chronic physical illness in group comparisons (6 studies, 42.8%), though two studies specifically only included participants with co-morbid cardiovascular disease. Use of a pre-determined cut-off value for cytokine levels was employed by three studies (21.4%) to ensure that cases with acute infection were excluded from the sample.

Many inflammatory markers exhibit a diurnal pattern of expression and are affected by consumption of food, thus time of day of sampling and whether the sample was taken in a fasted state are important factors to consider in analysing relative levels of cytokines[45]. However, time of day of sampling was only recorded in a minority of studies (6 studies, 42.8%), and the same number of studies recorded whether fasted samples were taken.

The overall quality of studies included in the review varied significantly, with Newcastle-Ottawa scale scores ranging from 2 to 9 (see Table 3). The area in which most studies were inadequate was in reporting non-response rate and detailing recruitment methods (see Table 3). Lowest quality studies were abstracts or dissertations, and two studies lacked control groups as only GAD patients were sampled (see Table4).

C-reactive protein

CRP is a critical early pro-inflammatory surveillance molecule involved in the activation of the complement system and both innate and adaptive immune systems[46]. We identified nine studies that investigated the association between GAD and CRP, comprising a total of 11,486 participants (see Table 5).

Four studies, involving 578 GAD patients and 4046 controls, provided sufficient information to conduct a meta-analysis of CRP levels in GAD[33,39,41,43] (see Figure 2). This was the only inflammatory marker for which meta-analysis was possible. Meta-analysis showed significantly raised CRP in GAD compared to controls (SMD 0.38, 95% CI 0.06-0.69; Z=2.36, p=0.02) (see Figure 2). However, there was a large and statistically significant degree of heterogeneity between studies (Chi2=12.0; df=3; p=0.007; $I^2 = 75\%$). Given the high heterogeneity and inclusion of less than 10 studies in the meta-analysis we did not have sufficient power to examine publication bias[47].

For peer review only

Five studies[30,33,39,41,43] (n=4669), one of which was conducted in 16 year olds[39] and two in participants with co-morbid heart disease[30], reported significantly higher CRP levels in participants with a diagnosis of GAD. The largest study[31] (n=5810) examining CRP in GAD examined CRP levels in children from baseline measurement aged 9-16 years to follow up aged 19-21.This was the only study to examine the longitudinal association between GAD and CRP, and found a bivariate association both cross-sectionally and over time between GAD and elevated CRP , however, this was accounted for by potential co-variates including BMI and medication use. The only study[40] to find an inverse correlation between CRP and GAD was conducted in non-smoking women from a longitudinal study in Finland and did not specify the numbers of participants with a diagnosis of GAD or group differences.

No difference was found in a cohort study (n=821) that used a combined inflammatory index consisting of CRP, IL-6 and TNF-α in 93 patients with a diagnosis of GAD and controls with a history of cardiovascular disease (CVD)[35]. Subgroup analysis examining differences in individual inflammatory markers was not reported[35].

We found two studies[36,43] (n= 196) that examined the association of severity of GAD symptoms with CRP level. One found a significant positive correlation between CRP level and GAD-7 scores[43], and the other reporting CRP differences in 70 GAD patients with and without a diagnosis of alexithymia found a significant association between higher CRP and suicidal ideation[36].

Although the meta-analysis and the majority of included studies reported raised CRP in GAD, there was wide variation in reporting and adjustment for important potential moderators, including co-morbid MDD, use of medications, assay used and time of day of blood collection, all of which likely contributed to the high degree of heterogeneity between studies. Of the nine studies to analyse CRP, four (44.4%) did not exclude or adjust for medication use by participants[30,35,39,40]. Co-morbid MDD was not adjusted for in analysis by two studies[31,35], one of which was included in the meta-analysis[31]. Only three of the nine studies reported time of sample collection[33,36,43] or whether this was in a fasted state[33,35,36], and though all studies utilised a similar assay method, different assay types were used in every study.

In summary, of the nine studies to have examined differences between GAD and controls, the majority reported raised CRP in GAD and meta-analysis found significantly raised CRP in GAD with a small effect size. However, there was wide variation in study methods including variable adjustment for mediators of inflammation such as co-morbid MDD, medication use and sampling methods. Only one study examined CRP in GAD longitudinally, reporting a bivariate association accounted for by health seeking behaviours[31].

Interleukins

Seven studies examined the association between interleukins and GAD (see Table 3). IL-6 is a mediator of T-cell and B-cell activation and induces acute phase proteins in hepatocytes,

For peer review only

among other functions[46]. Pharmacological blockade of IL-6 action is used to treat several auto-immune conditions including RA, and raised IL-6 has been associated with a number of psychiatric conditions including depression, schizophrenia and PTSD[46,48]. We found IL-6 was the most frequently measured interleukin, with five studies (n=2066) examining changes in GAD patients compared to controls[33–35,37,43]. The largest study investigated differences between 454 participants with a diagnosis of GAD and 556 controls from The Netherlands Study of Depression and Anxiety (NESDA) cohort[33]. Though analysis was conducted on anxiety disorders as a whole, mean difference in IL-6 in people with GAD compared to controls obtained through direct communication with the author showed significantly higher levels in GAD. However, it is unclear whether these differences remain significant after adjustment for group differences and no associations were found between IL-6 and participants who had all types of anxiety disorder[33]. Two studies[34,43] (n=165), one of which used saliva samples[34], reported significantly higher IL-6 in medication naïve participants with a diagnosis of GAD compared to age and sex matched healthy controls.

No difference was found in a combined inflammatory index consisting of CRP, IL-6 and TNF-α in a study of 93 patients with GAD and co-morbid ischaemic heart disease[35]. One case-controlled study[43] of 48 Chinese outpatients presenting for the first time with a diagnosis of GAD and 48 age, sex and education matched controls accounted for all results for IL-1α, 5, 8, 12p70. This study found significantly higher levels of IL-1α, -8 and -12p70 in GAD patients, in addition to higher levels of IL-1α and IL-8 with increased severity of GAD (as measured by GAD-7 scale), but did not account for chronic physical co-morbidities during recruitment or in analysis. Both IL-1α and IL-8 have pro-inflammatory functions as chemo-attractants for leukocytes and haematopoiesis, and have been targeted for treatments in a number of auto-immune conditions[46]. However, there was no association between GAD and IL-5, which is thought to predominantly mediate myeloid cell activation, and is a target of treatment in asthma[46]. The same study[43] also examined IL-2, which has a major role in T-cell mediated autoimmune and inflammatory conditions[46]. Results showed significantly higher IL-2 in GAD patients, however, this conflicted with results from a smaller study (n=24) that found no significant difference between medication naïve GAD patients and controls, though few details of the characteristics of participants, sampling or analysis were reported in this abstract[32].

One study that measured IL-1 using sputum analysis found significantly higher levels in GAD compared to controls in 69 participants recruited from the same Chinese hospital[34]. Though IL-1 is pro-inflammatory, there are differences in function dependent on the class of IL-1 protein measured which was not reported in this study[34].

IL-4 has several pro-inflammatory functions including immunoglobulin-E (Ig-E) class switching, expression of MHC class II and acts as a survival factor for T and B cells[46]. The only study to measure IL-4, found no differences between 54 GAD patients recruited from community mental health teams and primary care after controlling for age, sex, BMI, smoking, alcohol consumption and co-morbid depression[38]. This study[38] also investigated IL-10, which was the only cytokine with an anti-inflammatory function to be measured and is

For peer review only

involved in immunosuppression of T cell subsets and B cell immunoglobulin production. This found significantly lower levels of IL-10 (OR 0.35 $p = 0.003$) in GAD patients. However, this opposed findings from a smaller study (n=24) which reported significantly higher levels of IL-10 in GAD patients compared to controls, though it was not reported whether this association remained significant after controlling for group differences[32].

In summary, IL-6 was the most commonly measured interleukin raised in GAD compared to controls in the majority of studies, however, no study examined the longitudinal association with GAD. Other interleukins were examined by relatively few studies that examined small numbers cross-sectionally with mixed findings.

IFN-γ

IFN-γ has anti-viral roles including promoting cytotoxic activity, MHC class I and II upregulation, NK cell activation, and is a treatment target in inflammatory conditions such as Crohn's disease[46]. Three studies investigated IFN-γ levels in GAD (n= 330)[38,40,43]. The largest study (n=118) found higher IFN-γ in GAD patients from the UK that remained significant after adjustment for age, gender, BMI, smoking, alcohol and co-morbid depression, but did not adjust for anxiolytic medication use in analysis[38]. This finding was supported by a study of 96 participants which reported higher IFN-γ levels in GAD and a significant positive correlation between anxiety severity and IFN-γ[43]. Conflicting findings were reported by a Finnish study of 116 participants, which found significantly lower IFN-γ in GAD patients. However, the number of participants with a diagnosis of GAD, differences between groups and adjustment for potential confounders were not reported[40]. In summary, only a few small cross-sectional studies have examined differences in IFN-γ between GAD and control groups, and their findings were mixed.

TNF-α

TNF-α has a wide array of roles in host defence, including initiating a strong acute inflammatory response but limiting duration of inflammatory activation, and is the target of blocking monoclonal antibodies in the treatment of a wide array of autoimmune conditions including Crohn's disease and RA[46]. Six studies (n=2300) investigated TNF-α in GAD, with mixed findings. Three studies (n=303) found TNF-α significantly raised in GAD patients compared to controls[34,38,40]. However, the largest study to measure TNF-α (n=1010) found no difference between participants with GAD and controls, and no correlation between TNF-α and anxiety symptoms[33]. This finding was supported by a study of 93 patients with GAD and co-morbid ischaemic heart disease using a combined inflammatory index of CRP, IL-6 and TNF-α which reported no difference compared to controls[35]. In summary, though the majority of studies to measure differences in TNF-α between GAD and controls reported significantly raised levels, these comprised small cross-sectional studies and the largest study reported no difference.

Other cytokines

One study compared levels of the pro-inflammatory cytokines CCL-5, MCP-1 and SDF-1 in 120 medication naïve physically well patients with a diagnosis of GAD and co-morbid

For peer review only

personality disorder to 40 controls[42]. Significantly higher levels of MCP-1 and SDF-1 were reported in both men and women, and higher CCL-5 in men but not women with a diagnosis of GAD compared to controls[42].

Discussion

To our knowledge, this is the first systematic review and meta-analysis focusing on inflammatory cytokines in GAD. Using a range of databases we identified 14 studies, comprising 1188 participants with GAD and which measured 16 cytokines. We found significantly raised levels of CRP, IFN-γ and TNF-α in people with GAD compared to controls which were findings replicated in two or more studies. A further 10 pro-inflammatory cytokines were reported to be significantly raised in GAD in at least one study, however, 5/14 studies found no difference in at least one cytokine.

Despite substantial efforts to acquire data by contacting authors, it was only possible to conduct a meta-analysis of CRP. This identified significantly higher levels in GAD compared to controls with a small effect size (SMD: 0.38), though there was evidence of significant heterogeneity across studies ($I^2 = 75\%$). This effect size in CRP is greater than has been reported in other anxiety disorders (PTSD: SMD = -0.14)[24] or MDD (SMD= 0.14)[6], and is similar to that reported in schizophrenia (SMD= 0.45)[49]. Though we were only able to meta-analyse CRP, meta-analyses of different cytokines in other anxiety disorders have been conducted with larger effect sizes. A meta-analysis of inflammatory markers in PTSD identified 20 studies which reported increased interleukin 6 (IL-6), interleukin 1β (IL-1β), tumour necrosis factor-α (TNFα), and interferon γ (IFN γ) levels with effect sizes ranging from small (IFN γ: SMD 0.49) to large 1.42 (IL-1β: SMD 1.42)[24]. However, a systematic review and meta-analysis of pro-inflammatory cytokines in OCD identified 12 studies, and concluded that there was a significant reduction in IL-1β with moderate effect size (SMD: -0.60, $p<0.001$), and only IL-6 levels were significantly increased after subgroup analysis in medication-free adults with OCD[7]. It is unclear whether this profile of inflammatory marker changes would follow a similar pattern in GAD if future studies enabled further meta-analysis.

However, our findings should be interpreted with caution, due to the high heterogeneity among studies, low participant numbers and inconsistent reporting and adjustment for known confounding factors such as BMI, smoking, medication use and co-morbidities. It was not possible to analyse the cause of the degree of heterogeneity due to the paucity of studies. Other known mediators of inflammation[24] such as physical activity, raised blood pressure and genetics were not accounted for. Furthermore, reporting of GAD severity and duration of symptoms was generally poor, preventing detailed analysis of whether inflammatory markers predicted outcomes and quality of life. We also found limitations in inclusion of specific demographics of participants with GAD. For example, despite GAD in older adults being

For peer review only

prevalent and often treatment resistant[50,51,52], only two studies included participants over the age of 65, both of which only included patients with co-morbid ischaemic heart disease.

We are beginning to understand the interplay between cytokines, the immune system and mental health[1,53]. At a molecular level we are aware that pro-inflammatory cytokines, including IFN, IL-1β and TNF, can reduce the availability of monoamines by inducing expression of pre-synaptic reuptake pumps and inhibiting enzymes involved in monoamine synthesis[54], linking the monoamine theory of anxiety with inflammatory mechanisms. There is also a growing understanding of the relationship between systemic inflammation and the central nervous system (CNS)[1,55]. Microglial activation has been shown to be mediated by peripheral cytokines, and increased activation has been found in post-mortem studies of patients with MDD and schizophrenia[1]. No study we identified correlated inflammatory marker changes with in vivo microglial activation imaging in GAD and to our knowledge no research on post-mortem microglial changes in GAD has been conducted. Increased neuronal activity has also been shown to induce inflammatory and vascular changes in the brain, suggesting that psychological stress can not only be induced by inflammation but perpetuate chronic low grade inflammation seen in other vascular and neurodegenerative disorders[55]. Understanding interactions between the CNS and immune system, and identifying biomarkers of GAD offers potential for novel therapeutic approaches. The revolution of development of monoclonal-antibody therapies for inflammatory disorders[56] raises the possibility of repurposing these medications for trials in treatment resistant GAD if specific and consistent profiles of inflammatory biomarkers are identified.

However, it remains unclear as to whether inflammation plays a causal role in GAD[48,54]. For example, although IL-6 is a successful target for treatment in a number of auto-immune conditions and raised IL-6 is implicated in several psychiatric disorders, it also acts to reduce other pro-inflammatory cytokines such as TNF via negative feedback and is induced by physical exercise, hyperthermia, fasting, sleep deprivation and sunlight exposure without activation other pro-inflammatory cytokines[48]. This raises the question as to whether inflammation in GAD is a consequence rather than cause of symptoms. This will only be answered by large prospective longitudinal studies, better characterising the relationship between inflammation and GAD, and our review identified only one published study that examined inflammation in GAD patients longitudinally in a cohort of adolescents.

Conclusion

There is some preliminary evidence to suggest a raised inflammatory response in GAD, although it is unclear whether inflammatory cytokines play a role in aetiology. GAD remains a poorly studied area of psychiatric neuroinflammatory research compared to other mental illnesses such as MDD and schizophrenia. While we are a long way from using inflammatory cytokines as a biomarker or treatment target in GAD, current findings reflect inflammatory changes seen in other mental illnesses and highlight the importance of ongoing investigation of the role inflammation plays in the development and course of GAD. Further,

methodologically consistent, prospective, longitudinal studies examining the mechanisms and relationship between inflammation and GAD, while accounting for known mediators of cytokine production, are required.

Funding

This research received no specific grant from any funding body. HC and EA are supported by a NIHR Academic Clinical Fellowship. RG and RH are supported by the NIHR UCLH BRC

For peer review only

Table 1. Study & clinical characteristics

Study	Country	Study type	Inflammatory markers	N: GAD	N: Control	Standardised diagnostic assessment/critera	Anxiety measure	Age (years, SD): GAD	Age (years, SD): Control	% Female: GAD	% Female: Control	BMI: GAD	BMI: Control	Current smoking	Medicated	Physical health co-morbidities	Mental health co-morbidities
**Bankier et al. 2008**	US	Case-control	CRP	15	30	SCID, DSM IV	nr	nr	67.6 (12.7)	nr	33%	nr	nr	GAD: nr. Controls: 10%	nr	All participants had CVD. Excluded other conditions.	Excluded.
**Copeland et al. 2012**	US	Cohort, prospective	CRP	146	5664	Child and adolescent psychiatric assessment <16, Young adult psychiatric assessment >16. DSM IV criteria.	Total number of anxiety symptoms (range: 0-6)	14.21 All subjects (Odds or means ratio 1.12 (1.03-1.22) with GAD dx)		48.7% (Odds or means ratio 2.02 (1.04-3.92) with GAD dx)		22.37 (5.62) (Odds or means ratio 1.08 (1.05-1.11) with GAD dx)		Total sample: 13.5% (Odds/means ratio with GAD: 2.86)	30.2% 'use medication' (Odds/means ratio with GAD: 2.00)	34.7% had 'recent health ailments'.	Total sample: 39.9% co-morbid MDD.
**De Berardis et al. 2017**	Canada	Cross-sectional	CRP	70	no control	SCID, DSM IV	HAM-A (score >20 for inclusion)	28.2 (5.3)	-	51.40%	-	22.1 (1.67)	-	nr	Excluded	Excluded	Total sample: 44.3% of participants had alexithymia. Excluded other co-mordid mental illness
**Hoge et al. 2016**	US	RCT	TNF-α, IL-6	70	no control	SCID, DSM IV	nr	39.12	-	45.70%	-	nr	-	nr	Excluded	Excluded	GAD: 14.3% co-morbid MDD. Excluded other co-mordid mental illness
**Hou et al. 2017**	UK	Case-control	IL-4, IL-10, TNF-α, IFN-γ	54	64	MINI, DSM-IV & ICD-10 criteria	HADS, GAD-7 (score >10 for inclusion) .	35.06 (14.45)	25.75 (8.87)	34%	50%	24.84 (5.70)	22.45 (3.27)	GAD: 22% Controls: 34%	GAD: 67% use 'anxiolytic' medication. Excluded other medication use.	Excluded.	Excluded
**Khandaker et al. 2016**	UK	Cohort, prospective	CRP	26	3392	DAWBA. DSM-IV criteria	DAWBA	15.56 (0.24)	15.53 (0.31)	90%	52.30%	22.55 (3.52)	21.40 (3.63)	nr	nr	nr	GAD: 30.77% co-morbid MDD. Excluded other co-mordid mental illness.
**Korkelia et al. 2010**	Finland	Cross-sectional	CRP, TNF-α, IFN-γ	116		MINI	nr	nr	nr	100%	100%	All 25.3 (5.0)	nr	Excluded	nr	nr	nr
**Nayek et al 2016**	India	Case-control	CRP	50	50	ICD-10	nr	37.96 (10.7)	37.00 (12.08)	54%	23%	nr	nr	Excluded	Excluded if using HRT or OCP. Other medications not reported.	Excluded	nr
**Oglodek et al. 2015**	Poland	Case-control	SDF-1, CCL-5, MCP-1	120	40	DSM-V	nr	41.4 (3.5)	40.8 (3.1)	50%	nr	nr	nr	nr	Excluded	Excluded	All participants had co-morbid personality disorder. Excluded other co-mordid mental illness.
**Tang et al. 2017**	China	Case-control	CRP, IL-1α,, IL-2, IL-5, IL-6, IL-8, IL-12p70, IFN-γ, GM-CSF	48	48	MINI, DSM-IV	GAD-7, SAI, TAI	40.75 (12.21)	39.56 (10.06)	58.33%	64.17%	22.56 (2.73)	22.69 (2.63)	GAD: 29% . Controls: 23%	Excluded	Excluded acute illness. Chronic co-morbidities nr.	Excluded
**Tofani et al. 2015**	Italy	Case-control	IL-2, IL-10	14	10	MINI, DSM-IV	GAD-7	nr	nr	nr	nr	nr	nr	nr	Excluded	nr	Excluded
**Vogelzangs et al 2013**	Holland	Cohort	CRP, IL-6, TNF-α	454	556	CIDI, DSM criteria	BAI	Total sample: 41.8 (13.1)		66.90%		25.6 (5.1)		Total sample: 38.2%	nr	Total sample: 6.2% CVD, 4.9% diabetes, mean of 0.4 other chronic diseases.	Total sample: 58.4% co-morbid MDD. Excluded other co-mordid mental illness.
**Yang et al. 2017**	China	Case-control	IL-1, IL-4, TNF-α	28	41	MINI, DSM IV	HAM-A	55.1 (6.9)	55.9 (5.6)	53.60%	48.80%	22.0 (4.4)	22.5 (3.7)	GAD: 35.7%. Control: 24.4%	Excluded	Additional group with co-morbid asthma. Excluded other co-morbidities.	Excluded
**Zahm 2016**	US	Cohort, prospective	CRP, IL-6, TNF-α	93	728	CDIS, DSM IV	nr	68 (9.6)		17%		nr	nr	nr	nr	All patients had history of CVD.	GAD: 60.0% co-morbid MDD, 86.2% had lifetime history MDD.

not reported (nr), not applicable (-), sd: standard deviation, C-reactive protein (CRP), interleukin (IL-), tumour necrosis factor-α (TNF-α) , interferon-γ (IFN-γ), stromal derived factor-1 (SDF-1), monocyte chemoattractant protein-1 (MCP-1), chemokine C-C motif l
Mental Disorders (DSM), International classification of disesases (ICD), Computerised diagnostic interview schedule (CDIS),Composite Interview Diagnostic Instrument (CIDI), Mini-International Neuropsychiatric Interview (MINI), Structure clinical interview for D
(MADRS), Hamilton Anxiety Rating Scale (HAM-A), Hospital Anxiety and Depression Scale (HADS), Generalised Anxiety Disorder Assessment (GAD-7), State-Anxiety Inventory (SAI), Test Anxiety Inventory (TAI), Beck Anxiety Inventory (BAI), Cardiovascular disease (CVD), contraceptive pill (OCP), Hormone replacement therapy (HRT)

For peer review only

Table 2. Inflammatory marker sampling and analysis

Study	GAD	Control	Inflammatory markers	Nature of sample	Cross-sectional or longtidiunal	Time of day of sample	Fasted period before sample	Assay method	Assay sensitivity reported	Inflammatory marker cutoff used.	Confounding factors controlled for
**Bankier et al. 2008**	15	30	CRP	Blood	Cross-sectional	nr	nr	High sensitivity tubidometric immunoassay	Yes	CRP >3mg/l for significance	Age, sex, education, MDD, obesity, smoking history, type II diabetes mellitus, hypertension, hyperlipidaemia, other mental illness.
**Copeland et al. 2012**	146	5664	CRP	Whole blood spots	Longitudinal: Sampled aged 9-16, 19, and 21 years old.	nr	nr	Biotin-Streptavidin based Immunofluorometric system.	Yes	Excluded if >10 mg/l	Age, sex, race, SES, BMI, medication use, substance use, recent physical illness, chronic illness.
**De Berardis et al. 2017**	70	no control	CRP	Serum	Cross-sectional	7- 8.30am	10 hour fast	Highly sensitive nephelometric assay	Yes.	No	Age, sex, BMI, MDD, physical illness, other mental illness, medication use
**Hoge et al. 2016**	70	no control	TNF-α, IL-6	Plasma	Longitudinal: sampled pre- and post-psychological intervention	1-4.30pm	nr	nr	No	No	Age, sex, ethnicity, MDD, medication use, physical illness, other mental illness
**Hou et al. 2017**	54	64	IL-4, IL-10, TNF-α, IFN-γ	Serum	Cross-sectional	9-10am	nr	Multiplex ultra-sensitive immunoassay	Yes	No	Age, sex, BMI, smoking, alcohol consumption, MDD, physical illness, other mental illness
**Khandaker et al. 2016**	26	3392	CRP	Serum	Cross-sectional	nr	'Overnight'	Automated particle-enhanced immunoturbidimetric assay	No	Excluded if >10 mg/l	Age, sex, parental SES, ethnicity, maternal age at delivery, concurrent infection, family history of inflammatory disease, MDD.
**Korkelia et al. 2010**	116		CRP, TNF-α, IFN-γ	Blood	Cross-sectional	nr	nr	nr	No	No	BMI
**Nayek et al 2016**	50	50	CRP	Serum	Cross-sectional	nr	nr	Particle enhanced turbidimetric immunoassay technique	No	Excluded if 'raised ESR'	Age, sex, SES, religion, marital status, locality, BMI >30, physical illness
**Oglodek et al. 2015**	120	40	SDF-1, CCL-5, MCP-1	Plasma	Cross-sectional	7-9am	Fasted, duration nr.	ELISA	Yes	No	Sex, other mental illness, physical illness, substance misuse, smoking status, medication use.
**Tang et al. 2017**	48	48	CRP, IL-1α,, IL-2, IL-5, IL-6, IL-8, IL-12p70, IFN-γ, GM-CSF	Serum	Cross-sectional	9-10am	nr	ELISA	No	No	Age, sex, education, BMI, smoking status, alcohol consumption, acute physical illness, other mental illness, medication use.
**Tofani et al. 2015**	14	10	IL-2, IL-10	Plasma	Cross-sectional	nr	nr	Immunoenzymatic assay	No	No	Medication use.
**Vogelzangs et al 2013**	454	556	CRP, IL-6, TNF-α	Plasma	Cross-sectional	8-9am	'Overnight'	ELISA	Yes	No	Age, sex, education, smoking status, alcohol intake, physical activity, BMI, physical illness, medication use, MDD, other mental illness
**Yang et al. 2017**	28	41	IL-1, IL-4, IL-6, TNF-α	Saliva	Cross-sectional	nr	'Overnight'	ELISA	Yes	No	Age, sex, smoking status, BMI, medication use, physical illness, other mental illness
**Zahm 2016**	93	728	CRP, IL-6, TNF-α	Serum	Cross-sectional	No. (fasting, duration nr)	Fasted, duration nr.	ELISA	Yes	No	Age, sex, SES, BMI, illicit substance use, alcohol use, smoking status, physical activity, physical illness

nr (not reported), C-reactive protein (CRP), interleukin (IL-), tumour necrosis factor-α (TNF-α) , interferon-γ (IFN-γ), stromal derived factor-1 (SDF-1), monocyte chemoattractant protein-1 (MCP-1), chemokine C-C motif ligand 5 (CCL-5), granulocyte-macrophage co linked-immuno-sorbent assay (ELISA), erythrocyte sedimentation rate (ESR), mg (milligrams), l (litre), socioeconomic status (SES), body mass index (BMI), major depressive disorder (MDD)

For peer review only

Table 3. Summary inflammatory marker findings in GAD

Study	N: Controls	N: n with GAD	Finding
			**C-reactive protein (CRP)**
Bankier et al. 2008	30	15	↑ in GAD with co-morbid CVD compared to controls using a dichotomous outcome of CRP cut off score (CRP >3mg/l).
Copeland et al. 2012	5664	146	Longitudinal study in adolescents: ↑ bivariate association both cross-sectionally & over time between GAD & elevated CRP, but accounted for by medication use & BMI.
De Berardis et al. 2017	no control	70	↑ in GAD patients with co-morbid alexithymia and with increased suicidal ideation, no control group.
Khandaker et al. 2016	3392	26	↑ in 16 year olds with GAD compared to controls, remained ↑ after adjusting for co-variates.
Korkelia et al. 2010	116		↓ in non-smoking women with diagnosis of GAD compared to controls, however control group not described.
Nayek et al. 2016	50	50	↑ in GAD patients compared to controls.
Tang et al. 2017	48	48	↑ in GAD patients compared to controls and ↑ with increased severity of GAD.
Vogelzangs et al 2013	556	454	↑ in GAD patients compared to controls in unadjusted data obtained from author.
Zahm 2016	728	93	↔ between those with & without a current GAD diagnosis (p = 0.28) or with & without a lifetime GAD diagnosis, using a combined inflammatory index of CRP, IL-6 & TNF-α measurements.
			**Interleukin 1 (IL-1)**
Yang et al. 2017	41	28	↑ sputum IL-1 in patients aged 50-60 years old with GAD compared to controls.
			**Interleukin 1α (IL-1α)**
Tang et al. 2017	48	48	↑ IL-1α in GAD patients compared to controls and ↑ with increased severity of GAD.
			**Interleukin 2 (IL-2)**
Tang et al. 2017	48	48	↑ in GAD patients compared to controls (p <0.001) but ↔ with severity of GAD.
Tofani et al. 2015	10	14	↔ in GAD patients compared to controls.
			**Interleukin 4 (IL-4)**
Hou et al. 2017	64	54	↔ in GAD patients compared to controls.
			**Interleukin 5 (IL-5)**
Tang et al. 2017	48	48	↔ in GAD patients compared to controls, or association with severity of GAD.
			**Interleukin 6 (IL-6)**
Hoge et al. 2016	-	70	No control group: RCT of psychological intervention in GAD
Tang et al. 2017	48	48	↑ in GAD patients compared to controls & ↑ with increased severity of GAD.
Vogelzangs et al 2013	556	454	↑ in GAD patients compared to controls in unadjusted data obtained from author, but ↔ between IL-6 & GAD compared to other anxiety disorders.
Yang et al. 2017	41	28	↑ sputum in GAD patients aged 50-60 years old compared to controls.
Zahm 2016	728	93	↔ between those with &without a current GAD diagnosis or with & without a lifetime GAD diagnosis, using a combined inflammatory index of CRP, IL-6 & TNF-α measurements.
			**Interleukin 8 (IL-8)**
Tang et al. 2017	48	48	↑ in GAD patients compared to controls and ↑ with increased severity of GAD.
			**Interleukin 10 (IL-10)**
Hou et al. 2017	64	54	↓ in GAD patients compared to controls, which remained ↓ after adjustment for co-variates.
Tofani et al. 2015	10	14	↑ in GAD compared to controls.
			**Interleukin 12p70 (IL-12p70)**
Tang et al. 2017	48	48	↑ in GAD patients compared to controls but ↔ with severity of GAD.
			**Interferon gamma (IFN-γ)**
Hou et al. 2017	64	54	↑ in GAD patients compared to controls which remained ↑ after adjustment for co-variates.
Korkelia et al. 2010	116		↓ in non-smoking women with diagnosis of GAD compared to controls, however control group not described.
Tang et al. 2017	48	48	↑ in GAD patients compared to controls and ↑ with increased severity of GAD.
			**Tumour necrosis factor-alpha (TNF-α)**
Hoge et al. 2016	-	70	No control group: RCT of psychological intervention in GAD
Hou et al. 2017	64	54	↑ in GAD patients compared to controls which remained ↑ after adjustment co-variates.
Korkelia et al. 2010	116		↑ in non-smoking women with a diagnosis of GAD compared to controls, though control group was not described.
Vogelzangs et al 2013	556	454	↔ in GAD patients compared to controls, & ↔ between TNF-α & GAD compared to other anxiety disorders.
Yang et al. 2017	41	28	↑ sputum TNF-α in GAD patients aged 50-60 years old compared to controls.
Zahm 2016	728	93	↔ between those with & without a current GAD diagnosis or with & without a lifetime GAD diagnosis, using a combined inflammatory index of CRP, IL-6 & TNF-α measurements.
			**Chemokine C-C motif ligand 5 (CCL-5) / Regulated on activation, normal T cell expressed and secreted (RANTES)**
Oglodek et al. 2015	40	120	↑ in males with GAD & comorbid personality disorder compared to controls
			**Monocyte chemoattractant protein-1 (MCP-1)**
Oglodek et al. 2015	40	120	↑ in GAD & comorbid personality disorder compared to controls
			**Stromal derived factor-1 (SDF-1)**
Oglodek et al. 2015	40	120	↑ in GAD & comorbid personality disorder compared to controls
			**Granulocyte-macrophage colony-stimulating factor (GM-CSF)**
Tang et al. 2017	48	48	↑ in GAD patients compared to controls and ↑ with increased severity of GAD.

↑ = statistically significant increase in inflammatory marker in people with GAD compared to controls ($p< 0.05$), ↓ = statistically significant decrease in inflammatory marker in people with GAD compared to controls ($p< 0.05$), ↔ = no statistically significant difference in inflammatory marker in people with GAD compared to controls ($p> 0.05$). RCT = randomised controlled trial.

Table 4. Study quality assessment: Newcastle Ottawa scale

	Selection				Comparability	Exposure			
	Adequate case definition	Cases representative	Selection of Controls	Definition of Controls	Comparability of design & analysis	Ascertainment of exposure	Same method of ascertainment	Non-response rate	TOTAL stars:
**Bankier et al. 2008**	◊	-	◊	◊	◊◊	◊	◊	◊	*8*
**Copeland et al. 2012**	◊	◊	◊	◊	◊◊	◊	◊	-	*8*
**De Berardis et al. 2017**	◊	-	na	na	na	◊	na	-	*2*
**Hoge et al. 2016**	◊	-	na	na	na	◊	na	-	*2*
**Hou et al. 2017**	◊	◊	◊	◊	◊◊	◊	◊	-	*8*
**Khandaker et al. 2016**	◊	◊	◊	◊	◊◊	◊	◊	◊	*9*
**Korkelia et al. 2010**	◊	-	◊	-	◊	-	◊	-	*4*
**Nayek et al 2016**	◊	-	◊	-	◊◊	◊	◊	-	*6*
**Oglodek et al. 2015**	◊	-	◊	◊	◊◊	-	◊	-	*6*
**Tang et al. 2017**	◊	◊	◊	◊	◊◊	◊	◊	-	*8*
**Tofani et al. 2015**	◊	-	-	-	◊	◊	◊	-	*4*
**Vogelzangs et al 2013**	◊	◊	◊	◊	◊◊	◊	◊	◊	*9*
**Yang et al. 2017**	◊	◊	-	◊	◊◊	◊	◊	-	*7*
**Zahm 2016**	◊	-	-	-	◊◊	◊	◊	-	*5*

◊ = met criteria, - = did not meet criteria, na = not applicable

For peer review only

Table 5. Additional critical appraisal

	Type of publication	Unrepresentative recruitment methods	Unrepresentative demographics	Group differences reported	Adjusted for group differences
**Bankier et al. 2008**	Paper	Yes. Recruited from cardiology clinic.	Yes. Older cohort due to cardiac co-morbidity required.	nr	nr
**Copeland et al. 2012**	Paper	No.	Yes. Aged 9-21 only.	Yes	Yes
**De Berardis et al. 2017**	Paper	No	No (aged 18-45)	No control	No control
**Hoge et al. 2016**	Paper	Yes recruited by advert as part of parent RCT	No (aged >18)	No control	No control
**Hou et al. 2017**	Paper	No	No (aged 18-65)	Yes	Yes
**Khandaker et al. 2016**	Paper	No	Yes, aged 16 years old only	Yes	Yes
**Korkelia et al. 2010**	Abstract	Yes, recruited from existing study in Finland.	Yes. Non-smoking women only.	No	Yes (BMI only)
**Nayek et al 2016**	Paper	Yes, inpatients only	No, (aged 18-65)	Yes	Yes
**Oglodek et al. 2015**	Paper	Yes, co-morbid personality disorder	No	Yes	No
**Tang et al. 2017**	Paper	No	No, (aged 18-60)	Yes	Yes
**Tofani et al. 2015**	Abstract	Recruitment method not stated	nr	nr	nr
**Vogelzangs et al 2013**	Paper	No	No (aged 18-65)	Yes	Yes
**Yang et al. 2017**	Paper	No	Yes, (aged 50-60).	Yes	Yes
**Zahm 2016**	Dissertation	Yes, cardiology patients only.	Yes, (aged >50)	Yes	Yes

For peer review only

References

1. Réus GZ, Fries GR, Stertz L, et al. The role of inflammation and microglial activation in the pathophysiology of psychiatric disorders. *Neuroscience*. 2015. doi:10.1016/j.neuroscience.2015.05.018
2. Brown AS, Vinogradov S, Kremen WS, et al. Prenatal exposure to maternal infection and executive dysfunction in adult schizophrenia. *Am J Psychiatry*. 2009. doi:10.1176/appi.ajp.2008.08010089
3. Benros ME, Waltoft BL, Nordentoft M, et al. Autoimmune diseases and severe infections as risk factors for mood disorders: A nationwide study. *JAMA Psychiatry*. 2013. doi:10.1001/jamapsychiatry.2013.1111
4. O'dushlaine C, Rossin L, Lee PH, et al. Psychiatric genome-wide association study analyses implicate neuronal, immune and histone pathways. *Nat Neurosci*. 2015. doi:10.1038/nn.3922
5. Khandaker GM, Cousins L, Deakin J, Lennox BR, Yolken R, Jones PB. Inflammation and immunity in schizophrenia: Implications for pathophysiology and treatment. *The Lancet Psychiatry*. 2015. doi:10.1016/S2215-0366(14)00122-9
6. Howren MB, Lamkin DM, Suls J. Associations of depression with c-reactive protein, IL-1, and IL-6: A meta-analysis. *Psychosom Med*. 2009. doi:10.1097/PSY.0b013e3181907c1b
7. Gray SM, Bloch MH. Systematic review of proinflammatory cytokines in obsessive-compulsive disorder. *Curr Psychiatry Rep*. 2012. doi:10.1007/s11920-012-0272-0
8. Munkholm K, Vinberg M, Vedel Kessing L. Cytokines in bipolar disorder: A systematic review and meta-analysis. *J Affect Disord*. 2013. doi:10.1016/j.jad.2012.06.010
9. Meyer U, Feldon J. Neural basis of psychosis-related behaviour in the infection model of schizophrenia. *Behav Brain Res*. 2009. doi:10.1016/j.bbr.2008.12.022
10. Goldsmith DR, Rapaport MH, Miller BJ. A meta-analysis of blood cytokine network alterations in psychiatric patients: Comparisons between schizophrenia, bipolar disorder and depression. In: *Molecular Psychiatry*. ; 2016. doi:10.1038/mp.2016.3
11. Rao JS, Harry GJ, Rapoport SI, Kim HW. Increased excitotoxicity and neuroinflammatory markers in postmortem frontal cortex from bipolar disorder patients. *Mol Psychiatry*. 2010. doi:10.1038/mp.2009.47
12. Khairova RA, Machado-Vieira R, Du J, Manji HK. A potential role for pro-inflammatory cytokines in regulating synaptic plasticity in major depressive disorder. *Int J Neuropsychopharmacol*. 2009. doi:10.1017/S1461145709009924
13. Kubera M, Obuchowicz E, Goehler L, Brzeszcz J, Maes M. In animal models, psychosocial stress-induced (neuro)inflammation, apoptosis and reduced neurogenesis are associated to the onset of depression. *Prog Neuro-Psychopharmacology Biol Psychiatry*. 2011. doi:10.1016/j.pnpbp.2010.08.026
14. Furtado M, Katzman MA. Neuroinflammatory pathways in anxiety, posttraumatic stress, and obsessive compulsive disorders. *Psychiatry Res*. 2015. doi:10.1016/j.psychres.2015.05.036
15. Andlin-Sobocki P, Wittchen HU. Cost of anxiety disorders in Europe. *Eur J Neurol*. 2005. doi:10.1111/j.1468-1331.2005.01196.x
16. Baxter AJ, Scott KM, Vos T, Whiteford HA. Global prevalence of anxiety disorders: A systematic review and meta-regression. *Psychol Med*. 2013. doi:10.1017/S003329171200147X
17. Bystritsky A. Treatment-resistant anxiety disorders. *Mol Psychiatry*. 2006. doi:10.1038/sj.mp.4001852
18. Bruce SE, Yonkers KA, Otto MW, et al. Influence of psychiatric comorbidity on recovery and recurrence in generalized anxiety disorder, social phobia, and panic disorder: A 12-year prospective study. *Am J Psychiatry*. 2005. doi:10.1176/appi.ajp.162.6.1179
19. Baldwin D, Woods R, Lawson R, Taylor D. Efficacy of drug treatments for generalised anxiety disorder: Systematic review and meta-analysis. *BMJ*. 2011. doi:10.1136/bmj.d1199
20. Cuijpers P, Sijbrandij M, Koole S, Huibers M, Berking M, Andersson G. Psychological treatment of

For peer review only

generalized anxiety disorder: A meta-analysis. *Clin Psychol Rev*. 2014. doi:10.1016/j.cpr.2014.01.002
21. Härter MC, Conway KP, Merikangas KR. Associations between anxiety disorders and physical illness. *Eur Arch Psychiatry Clin Neurosci*. 2003. doi:10.1007/s00406-003-0449-y
22. Roy-Byrne PP, Davidson KW, Kessler RC, et al. Anxiety disorders and comorbid medical illness. *Gen Hosp Psychiatry*. 2008. doi:10.1016/j.genhosppsych.2007.12.006
23. Uguz F, Akman C, Kucuksarac S, Tufekci O. Anti-tumor necrosis factor-α therapy is associated with less frequent mood and anxiety disorders in patients with rheumatoid arthritis. *Psychiatry Clin Neurosci*. 2009. doi:10.1111/j.1440-1819.2008.01905.x
24. Passos IC, Vasconcelos-Moreno MP, Costa LG, et al. Inflammatory markers in post-traumatic stress disorder: A systematic review, meta-analysis, and meta-regression. *The Lancet Psychiatry*. 2015. doi:10.1016/S2215-0366(15)00309-0
25. Michopoulos V, Powers A, Gillespie CF, Ressler KJ, Jovanovic T. Inflammation in Fear-and Anxiety-Based Disorders: PTSD, GAD, and beyond. *Neuropsychopharmacology*. 2017. doi:10.1038/npp.2016.146
26. Liberati A, Altman DG, Tetzlaff J, et al. The PRISMA statement for reporting systematic reviews and meta-analyses of studies that evaluate health care interventions: explanation and elaboration. In: *Journal of Clinical Epidemiology*. ; 2009. doi:10.1016/j.jclinepi.2009.06.006
27. Spitzer R, Williams J, Gibbon M, First M. Structured Clinical Interview for DSM-IV. In: *Encyclopedia of Behavioral Medicine*. ; 1994. doi:10.1007/978-1-4419-1005-9_66
28. Wells G, Shea B, O'Connell D, et al. *The Newcastle-Ottawa Scale (NOS) for Assessing the Quality of Nonrandomized Studies in Meta-Analyses.*; 2013. doi:10.2307/632432
29. Higgins JPT. Measuring inconsistency in meta-analyses. *BMJ*. 2003. doi:10.1136/bmj.327.7414.557
30. Bankier B, Barajas J, Martinez-Rumayor A, Januzzi JL. Association between C-reactive protein and generalized anxiety disorder in stable coronary heart disease patients. *Eur Heart J*. 2008;29(18):2212-2217. doi:10.1093/eurheartj/ehn326
31. Copeland WE, Shanahan L, Worthman C, Angold A, Costello EJ. Generalized anxiety and C-reactive protein levels: A prospective, longitudinal analysis. *Psychol Med*. 2012;42(12):2641-2650. doi:10.1017/S0033291712000554
32. Tofani T, Pallanti S, Di Cesare Mannelli L, Zanardelli M, Ghelardini C. An immunologic profile study in drug-naive generalized anxiety non depressed patients: A pilot study. *Eur Neuropsychopharmacol*. 2015;25.
33. Vogelzangs N, Beekman ATF, de Jonge P, Penninx BWJH. Anxiety disorders and inflammation in a large adult cohort. *Transl Psychiatry*. 2013;3:e249. doi:10.1038/tp.2013.27
34. Yang CJ, Liu D, Du YJ, Xu ZS, Shi SX. The pro-inflammatory cytokines, salivary cortisol and alpha-amylase are associated with generalized anxiety disorder (GAD) in patients with asthma. *Neurosci Lett*. 2017;656:15-21. doi:10.1016/j.neulet.2017.07.021
35. Zahm JL. Generalized anxiety disorder and inflammatory biomarkers in coronary heart disease: Sex-specific effects. *Diss Abstr Int Sect B Sci Eng*. 2018;78(12).
36. De Berardis D, Serroni N, Campanella D, et al. Alexithymia, suicide ideation, C-Reactive Protein, and serum lipid levels among outpatients with generalized anxiety disorder. *Arch Suicide Res*. 2017;21(1):100-112. doi:10.1080/13811118.2015.1004485
37. Hoge EA, Bui E, Palitz SA, et al. The effect of mindfulness meditation training on biological acute stress responses in generalized anxiety disorder. *Psychiatry Res*. 2017:No. doi:10.1016/j.psychres.2017.01.006
38. Hou R, Garner M, Holmes C, et al. Peripheral inflammatory cytokines and immune balance in Generalised Anxiety Disorder: Case-controlled study. *Brain Behav Immun*. 2017;62:212-218. doi:10.1016/j.bbi.2017.01.021
39. Khandaker GM, Jones PB, Zammit S, Lewis G. Association between serum C-reactive protein and DSM-IV generalized anxiety disorder in adolescence: Findings from the ALSPAC cohort. *Neurobiol Stress*. 2016;4:55-61. doi:10.1016/j.ynstr.2016.02.003
40. Korkeila J, Runsten S, Ollikainen S, Korkeila K. Generalized anxiety disorder and immunity markers in a stratified population sample. *Eur Psychiatry*. 2010;25. doi:10.1016/S0924-9338%2810%2971560-1

For peer review only

41. Nayek S, Ghosh S. A Comparative Study Of Serum C-Reactive Protein In Patients With Generalised Anxiety Disorder And Depression. *Indian J Psychiatry*. 2017;59(6, 2):S217.
42. Oglodek EA, Szota AM, Araszkiewicz A, Just MJ, Mos DM. The MCP-1, CCL-5 and SDF-1 chemokines as pro-inflammatory markers in generalized anxiety disorder and personality disorders. *Pharmacol Reports*. 2015;67(1):85-89. doi:10.1016/j.pharep.2014.08.006
43. Tang Z, Ye G, Chen X, et al. Peripheral proinflammatory cytokines in Chinese patients with generalised anxiety disorder. *J Affect Disord*. 2018;225:593-598. doi:10.1016/j.jad.2017.08.082
44. Kitahara CM, Trabert B, Katki HA, et al. Body mass index, physical activity, and serum markers of inflammation, immunity, and insulin resistance. *Cancer Epidemiol Biomarkers Prev*. 2014. doi:10.1158/1055-9965.EPI-14-0699-T
45. Petrovsky N, McNair P, Harrison LC. Diurnal rhythms of pro-inflammatory cytokines: Regulation by plasma cortisol and therapeutic implications. *Cytokine*. 1998. doi:10.1006/cyto.1997.0289
46. Akdis M, Aab A, Altunbulakli C, et al. Interleukins (from IL-1 to IL-38), interferons, transforming growth factor β, and TNF-α: Receptors, functions, and roles in diseases. *J Allergy Clin Immunol*. 2016. doi:10.1016/j.jaci.2016.06.033
47. Sterne JAC, Sutton AJ, Ioannidis JPA, et al. Recommendations for examining and interpreting funnel plot asymmetry in meta-analyses of randomised controlled trials. *BMJ*. 2011. doi:10.1136/bmj.d4002
48. Raison CL, Knight JM, Pariante C. Interleukin (IL)-6: A good kid hanging out with bad friends (and why sauna is good for health). *Brain, Behavior, and Immunity*. 2018.
49. Miller BJ, Culpepper N, Rapaport MH. C-reactive protein levels in schizophrenia: A review and meta-analysis. *Clin Schizophr Relat Psychoses*. 2014. doi:10.3371/CSRP.MICU.020813
50. Byers AL, Yaffe K, Covinsky KE, Friedman MB, Bruce ML. High occurrence of mood and anxiety disorders among older adults: The National Comorbidity Survey Replication. *Arch Gen Psychiatry*. 2010. doi:10.1001/archgenpsychiatry.2010.35
51. Wolitzky-Taylor KB, Castriotta N, Lenze EJ, Stanley MA, Craske MG. Anxiety disorders in older adults: A comprehensive review. *Depress Anxiety*. 2010. doi:10.1002/da.20653
52. Wetherell JL, Liu L, Patterson TL, et al. Acceptance and Commitment Therapy for Generalized Anxiety Disorder in Older Adults: A Preliminary Report. *Behav Ther*. 2011. doi:10.1016/j.beth.2010.07.002
53. Felger JC, Lotrich FE. Inflammatory cytokines in depression: Neurobiological mechanisms and therapeutic implications. *Neuroscience*. 2013. doi:10.1016/j.neuroscience.2013.04.060
54. Miller AH, Raison CL. The role of inflammation in depression: From evolutionary imperative to modern treatment target. *Nat Rev Immunol*. 2016. doi:10.1038/nri.2015.5
55. Xanthos DN, Sandkühler J. Neurogenic neuroinflammation: Inflammatory CNS reactions in response to neuronal activity. *Nat Rev Neurosci*. 2014. doi:10.1038/nrn3617
56. Reichert JM, Rosensweig CJ, Faden LB, Dewitz MC. Monoclonal antibody successes in the clinic. *Nat Biotechnol*. 2005. doi:10.1038/nbt0905-1073

For peer review only

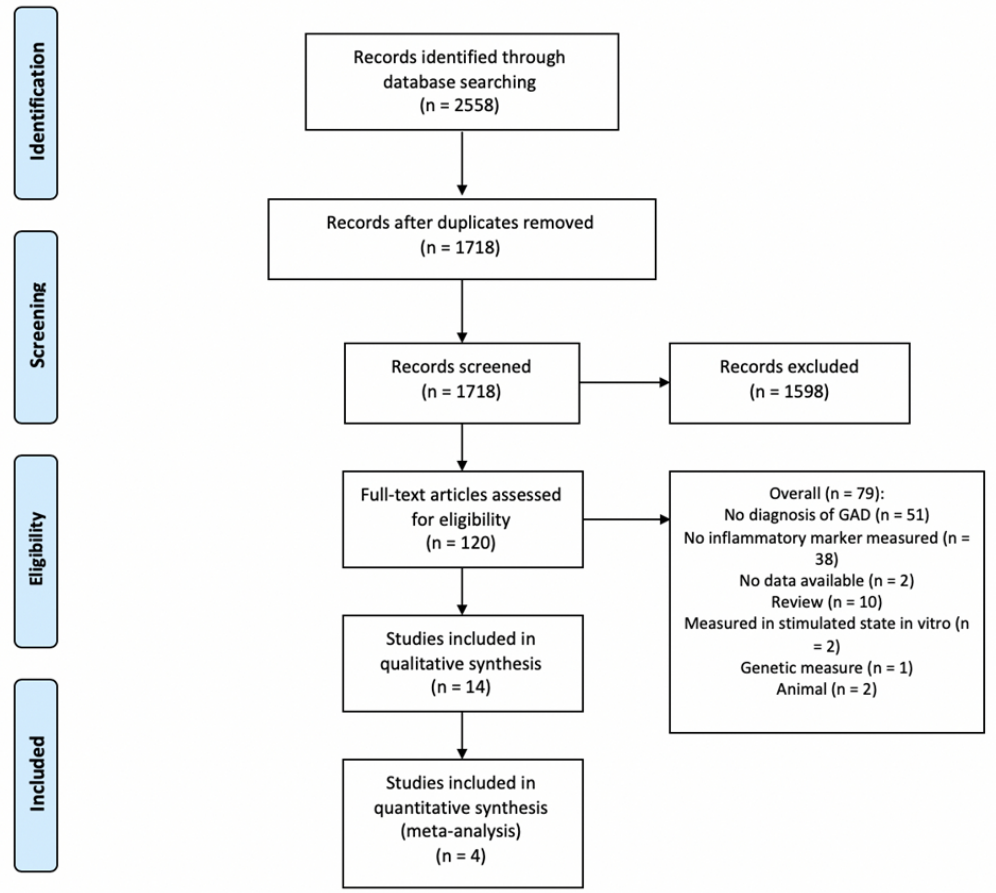

Figure 1. Flow of studies in the systematic review and meta-analysis.

Study or Subgroup	GAD Mean	GAD SD	GAD Total	Control Mean	Control SD	Control Total	Weight	Std. Mean Difference IV, Random, 95% CI
Khandaker 2010	0.91	2.97	26	0.39	10.1	3392	22.9%	0.05 [−0.33, 0.44]
Nayek 2016	0.29	0.14	50	0.2	0.1	50	22.1%	0.73 [0.33, 1.14]
Tang 2017	1.19	0.8	48	0.68	0.7	48	21.9%	0.67 [0.26, 1.08]
Vogelzangs 2013	3.1	4.9	454	2.3	4.4	556	33.1%	0.17 [0.05, 0.30]
**Total (95% CI)**			**578**			**4046**	**100.0%**	**0.38 [0.06, 0.69]**

Heterogeneity: $Tau^2 = 0.07$; $Chi^2 = 12.00$, df = 3 (P = 0.007); $I^2 = 75\%$
Test for overall effect: Z = 2.36 (P = 0.02)

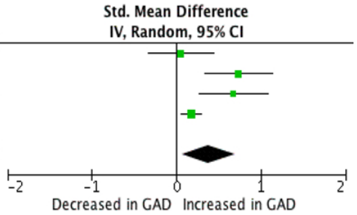

Figure 2. Forest plot of random effects meta-analysis of CRP levels in GAD vs controls.

PRISMA 2009 Checklist

Section/topic	#	Checklist item	Reported on page #
**TITLE**			
Title	1	Identify the report as a systematic review, meta-analysis, or both.	1
**ABSTRACT**			
Structured summary	2	Provide a structured summary including, as applicable: background; objectives; data sources; study eligibility criteria, participants, and interventions; study appraisal and synthesis methods; results; limitations; conclusions and implications of key findings; systematic review registration number.	2
**INTRODUCTION**			
Rationale	3	Describe the rationale for the review in the context of what is already known.	2-3
Objectives	4	Provide an explicit statement of questions being addressed with reference to participants, interventions, comparisons, outcomes, and study design (PICOS).	3-4
**METHODS**			
Protocol and registration	5	Indicate if a review protocol exists, if and where it can be accessed (e.g., Web address), and, if available, provide registration information including registration number.	3-4
Eligibility criteria	6	Specify study characteristics (e.g., PICOS, length of follow-up) and report characteristics (e.g., years considered, language, publication status) used as criteria for eligibility, giving rationale.	4
Information sources	7	Describe all information sources (e.g., databases with dates of coverage, contact with study authors to identify additional studies) in the search and date last searched.	4
Search	8	Present full electronic search strategy for at least one database, including any limits used, such that it could be repeated.	4
Study selection	9	State the process for selecting studies (i.e., screening, eligibility, included in systematic review, and, if applicable, included in the meta-analysis).	4
Data collection process	10	Describe method of data extraction from reports (e.g., piloted forms, independently, in duplicate) and any processes for obtaining and confirming data from investigators.	4
Data items	11	List and define all variables for which data were sought (e.g., PICOS, funding sources) and any assumptions and simplifications made.	4
Risk of bias in individual studies	12	Describe methods used for assessing risk of bias of individual studies (including specification of whether this was done at the study or outcome level), and how this information is to be used in any data synthesis.	5
Summary measures	13	State the principal summary measures (e.g., risk ratio, difference in means).	5
Synthesis of results	14	Describe the methods of handling data and combining results of studies, if done, including measures of consistency (e.g., I^2) for each meta-analysis.	5

For peer review only

PRISMA 2009 Checklist

Section/topic	#	Checklist item	Reported on page #
Risk of bias across studies	15	Specify any assessment of risk of bias that may affect the cumulative evidence (e.g., publication bias, selective reporting within studies).	4-5
Additional analyses	16	Describe methods of additional analyses (e.g., sensitivity or subgroup analyses, meta-regression), if done, indicating which were pre-specified.	5
**RESULTS**			
Study selection	17	Give numbers of studies screened, assessed for eligibility, and included in the review, with reasons for exclusions at each stage, ideally with a flow diagram.	5
Study characteristics	18	For each study, present characteristics for which data were extracted (e.g., study size, PICOS, follow-up period) and provide the citations.	5
Risk of bias within studies	19	Present data on risk of bias of each study and, if available, any outcome level assessment (see item 12).	5-6
Results of individual studies	20	For all outcomes considered (benefits or harms), present, for each study: (a) simple summary data for each intervention group (b) effect estimates and confidence intervals, ideally with a forest plot.	6
Synthesis of results	21	Present results of each meta-analysis done, including confidence intervals and measures of consistency.	6
Risk of bias across studies	22	Present results of any assessment of risk of bias across studies (see Item 15).	5-6
Additional analysis	23	Give results of additional analyses, if done (e.g., sensitivity or subgroup analyses, meta-regression [see Item 16]).	na
**DISCUSSION**			
Summary of evidence	24	Summarize the main findings including the strength of evidence for each main outcome; consider their relevance to key groups (e.g., healthcare providers, users, and policy makers).	6-11
Limitations	25	Discuss limitations at study and outcome level (e.g., risk of bias), and at review-level (e.g., incomplete retrieval of identified research, reporting bias).	6-11
Conclusions	26	Provide a general interpretation of the results in the context of other evidence, and implications for future research.	11-12
**FUNDING**			
Funding	27	Describe sources of funding for the systematic review and other support (e.g., supply of data); role of funders for the systematic review.	12

From: Moher D, Liberati A, Tetzlaff J, Altman DG, The PRISMA Group (2009). Preferred Reporting Items for Systematic Reviews and Meta-Analyses: The PRISMA Statement. PLoS Med 6(7): e1000097. doi:10.1371/journal.pmed1000097

For more information, visit: **www.prisma-statement.org**.

For peer review only - http://bmjopen.bmj.com/site/about/guidelines.xhtml

BMJ Open

A systematic review and meta-analysis of the association between peripheral inflammatory cytokines and generalised anxiety disorder

Journal:	*BMJ Open*
Manuscript ID	bmjopen-2018-027925.R1
Article Type:	Research
Date Submitted by the Author:	03-Apr-2019
Complete List of Authors:	Costello, Harry; University College London Division of Psychiatry,<br>Gould, Rebecca; University College London Division of Psychiatry<br>Abrol, Esha; University College London Division of Psychiatry<br>Howard, R; University College London, Division of Psychiatry
**Primary Subject Heading**:	Mental health
Secondary Subject Heading:	Immunology (including allergy)
Keywords:	Anxiety disorders < PSYCHIATRY, Immunology < BASIC SCIENCES, PSYCHIATRY

SCHOLARONE™ Manuscripts

A systematic review and meta-analysis of the association between peripheral inflammatory cytokines and generalised anxiety disorder

H. Costello[1], R.L. Gould[1], E. Abrol, R.J. Howard[1]

[1] Division of Psychiatry, University College London, London W1T 7NF, United Kingdom

* Corresponding author: Dr. Harry Costello (mailto:harry.costello@ucl.ac.uk)

4362 words (excluding abstract: 300 words)

For peer review only

Abstract

Objective: Inflammation has been implicated in the aetiology of mental illness. We conducted the first systematic review and meta-analysis of the association between peripheral markers of inflammation and generalised anxiety disorder (GAD).

Design: Systematic review and meta-analysis of studies measuring peripheral cytokine levels in people with GAD compared to controls.

Data sources: MEDLINE(1950–), EMBASE(1947-), PsycINFO(1872–) and Web of Science(1945–) databases up until January 2018.

Eligibility criteria: Primary, quantitative research studies of people with a diagnosis of GAD assessed using a standardised clinical interview that measured peripheral inflammatory markers.

Data extraction and synthesis: Two independent reviewers extracted data and assessed study quality. Meta-analysis using a random-effects model was conducted for individual cytokines where data from three or more studies were available.

Results: 14/1718 identified studies met inclusion criteria, comprising 1188 patients with GAD and 10,623 controls. In total 16 cytokines were evaluated. Significantly raised levels of CRP, IFN-γ and TNF-α were reported in GAD patients compared to controls in two or more studies. Ten further pro-inflammatory cytokines were reported to be significantly raised in GAD in at least one study. However, 5/14 studies found no difference in levels of at least one cytokine. Only CRP studies reported sufficient data for meta-analysis. CRP was significantly higher in people with GAD compared to controls, with a small effect size (Cohen's d = 0.38, 0.06-0.69), comparable to that reported in schizophrenia. However, heterogeneity was high (I^2= 75%), in keeping with meta-analyses of inflammation in other psychiatric conditions and reflecting differences in participant medication use, co-morbid depression and cytokine sampling methodology.

Conclusion: There is preliminary evidence to suggest an inflammatory response in GAD, but it remains unclear whether inflammatory cytokines play a role in aetiology. GAD remains a poorly studied area of neuroinflammation compared to other mental disorders and further longitudinal studies are required.

For peer review only

Strengths and limitations of this study

- This is the first study to conduct a comprehensive systematic review and meta-analysis of peripheral inflammatory markers in generalised anxiety disorder.
- A wide range of databases were searched, and a large number of papers screened for inclusion in the study, 14 of which were subjected to quality assessment and detailed critical appraisal.
- It was only possible to conduct a meta-analysis of C-reactive protein, and it was not possible to examine publication bias due to the limited number of studies identified for inclusion in the meta-analysis.
- The high levels of heterogeneity across studies mean that findings should be interpreted with caution.

For peer review only - http://bmjopen.bmj.com/site/about/guidelines.xhtml

Introduction

There is growing evidence for immune mediated pathogenic mechanisms in several psychiatric disorders with discrete profiles of inflammatory mechanisms[1]. Epidemiological evidence has shown an increased risk of mood disorders and psychosis in people with a history of severe infection or autoimmune conditions[2,3]. This has been supported by genome wide association studies implicating multiple immune signalling pathways[4], and altered profiles of pro-inflammatory cytokines and acute phase reactants in schizophrenia[5], depression[6], obsessive compulsive disorder (OCD)[7] and bipolar disorder[8]. However, the relationship between inflammation and mental illness remains poorly understood and controversial, with a number of proposed potential neuropathological mechanisms[9,10], including changes in microglial function[1], glutamatergic excitotoxicity[11], synaptic plasticity[12] and reduced hippocampal neurogenesis[13].

Despite increasing interest in the role of inflammation in mental illness, relatively little research has focused on potential associations with anxiety disorders[14]. These are common, with an estimated lifetime prevalence of 7.3% to 28.8%, are associated with substantial functional impairment and are estimated to cost between 42-47 billion dollars to the U.S economy each year[15,16]. However, only 60% of patients are thought to respond to pharmacological and psychological treatments and understanding of the underlying pathophysiological mechanisms of anxiety disorders remains poor[17].

Generalised anxiety disorder (GAD) is the most common anxiety disorder, with a degree of associated disability equivalent to that of major depressive disorder (MDD)[18]. Despite psychopharmacological[19] and psychological[20] treatments showing effectiveness in GAD, 42% of people living with GAD experience ongoing symptoms after 12 years and half of remitted patients experience recurrence[18]. GAD is more prevalent in those with inflammatory conditions such as rheumatoid arthritis (RA)[21,22], with case series studies suggesting symptoms are less common with immune modulating treatment targeting specific inflammatory cytokines[23]. The chronic clinical course and relatively high probability of recurrence in GAD, in addition to preliminary evidence of an inflammatory component in other anxiety disorders[7,24], suggest that inflammation could be an important neurobiological mechanism in the aetiology of this disorder.

To date, two previous reviews of inflammatory biomarkers in GAD have been conducted. Of these, however, one was a narrative review [25] and the other was restricted to literature published within the last decade [14], and with a focus on all anxiety disorders. Both reviews reported that there was preliminary evidence for inflammatory changes in GAD. However, only three studies were identified by the systematic review reporting cytokine changes in GAD and no meta-analysis was performed. No study to date has conducted a comprehensive systematic review and meta-analysis of all current literature focusing on GAD or commented on the longitudinal association between inflammation and GAD.

For peer review only - http://bmjopen.bmj.com/site/about/guidelines.xhtml

We aimed to systematically review the cross-sectional and longitudinal associations between inflammatory biomarkers and GAD, and perform the first meta-analysis of inflammatory biomarkers in GAD.

Method

We conducted a systematic review of studies that had included people with GAD who had undergone peripheral cytokines measurement and a between group meta-analysis of cytokine levels in people with GAD compared to controls. We conducted the study according to Preferred Reporting Items for Systematic Reviews and Meta-analyses (PRISMA) guidelines[26].

We searched MEDLINE (1950–), EMBASE (1947-), PsycINFO (1872–) and Web of Science (1945–) databases up until January 2018. Reference lists of eligible studies were then searched for further ones that met eligibility criteria.

Our search terms (see appendix 1 for further details) were: (inflammat* or cytokine or interferon or IFN or interleukin or "translocator protein" or TSPO or "tumour necrosis factor" or "tumor necrosis factor" or TNF or IL-1 or IL-2 or IL-4 or IL-7 or IL-6 or IL-8 or IL-10 or migroglia or t-cell or lymphocyte or "C-reactive protein" or "C reactive protein" or CRP or "acute phase protein" or "fibrinogen") and ("generalised anxiety disorder" or "generalized anxiety disorder" or GAD or worry).

We included primary, quantitative research studies (including unpublished theses and dissertations), written in any language, that included people with a diagnosis of GAD assessed using standardised clinical interview (e.g. Structured Clinical Interview for DSM[27]) or standardised psychometric instruments. Studies reported cross-sectional or longitudinal data in clinical or community populations. Cross-sectional studies measured inflammatory biomarker concentrations in anxious people versus non-anxious healthy controls, while longitudinal studies measured inflammatory biomarker concentrations at baseline and anxiety scores at follow-up. Inflammatory markers were measured in the unstimulated state (no antigen induced stimulation of cytokine production) and sampled from peripheral blood, CSF, or saliva at any time of day. Exclusion criteria included studies with less than 5 participants, studies in animals and studies where subjects were participants in the treatment arms of clinical trials.

Patient and public involvement

There was no patient or public involvement in the study.

Data extraction and quality assessment

Data were extracted and quality assessed for all studies that met eligibility criteria by two independent raters (HC, EA) with disagreements settled by consensus and discussion. For each cytokine, we extracted the means, variance estimates or 95% confidence intervals (CIs) and sample size for GAD and control groups. We also extracted demographic data (e.g. age,

For peer review only - http://bmjopen.bmj.com/site/about/guidelines.xhtml

sex) and clinical data (e.g. medication use, co-morbid depression, severity) where available. Authors were contacted for further information, where necessary.

Risk of bias and study quality were evaluated using the Newcastle–Ottawa Quality Assessment Scale[28]. Other potential confounding factors (including assay type and sensitivity, inflammatory marker analysis and recruitment methods) were also examined to allow more detailed bias and quality analysis of studies.

Strategy for data synthesis

Separate meta-analyses were performed for individual biomarkers in GAD versus controls if sufficient data were available from a minimum of three studies. Due to different measurement methods and anticipated high heterogeneity, we estimated a standardised mean difference (SMD) for each inflammatory marker, and used a random effects model for meta-analysis, conducted using Revman 5. Heterogeneity across studies was quantified with the I^2 statistic, with a value of 25% typically regarded as low, 50% as medium, and 75% as high[29]. If studies were longitudinal or trials of interventions with multiple data collection points, we examined baseline data only to avoid skewed meta-analysis from inclusion of more than one effect size from the same study.

Results

Systematic review

We identified 1718 papers, excluded 1598 of these by titles and abstracts, and retrieved the remaining 120 papers, of which 14 met eligibility criteria and were included in the final systematic review (see Figure 1). The primary reasons for rejection were that no diagnosis of GAD was recorded or no inflammatory marker was measured.

Characteristics of the 14 included studies are shown in Tables 1 and 2. Studies comprised a total of 1188 people with a diagnosis of GAD and 10,623 controls, with a further 116 participants from a study that did not report GAD and control group sizes.

In total, 16 different cytokines were evaluated (see Table 2) C-reactive protein (CRP) (9/14 studies, 64.2%), tumour necrosis factor-α (TNF-α) (6/14 studies, 42.9%), IL-6 (5/14 studies, 35.7%) and interferon-γ (IFN-γ) (3/14 studies, 21.4%) were most commonly studied. All other cytokines were only analysed in two or less studies.

Twelve studies (85.7%) reported the assay method used, all of which were versions of an enzyme-linked immunosorbent assay (ELISA) or enzyme immunoassay (EIA). However, only 7 studies (50%) reported assay sensitivity. All but one study used blood component samples to assess inflammatory marker levels, with the most common sample type being serum (N=6, 42.9%) and plasma (N=4, 28.6%).

For peer review only - http://bmjopen.bmj.com/site/about/guidelines.xhtml

Risk of bias and quality in individual studies

All included studies had adequate case definition, with participants meeting diagnostic criteria for GAD according to DSM or ICD, with 12 studies (85.7%) using a structured clinical interview for assessment (see Table 1).

Most (71.4%) studies included people aged 18-65, but two studies (14.3%) only included participants over the age of 50 and a further two studies (14.3%) used adolescent participant cohorts. The majority (78.6%) of studies accounted for age and sex differences in their analyses. Only 8/14 studies (57.1%) recorded participants' body mass index (BMI) which is known to correlate with inflammation, and only half of these accounted for BMI differences in analysis of group differences[30] (see Table 2).

Use of psychotropic medication and presence of comorbid major depressive disorder are important moderators of inflammation in other psychiatric disorders[24]. Six studies (42.8%) excluded patients who used psychiatric or other immune-modulating medication, though only two studies (4.3%) reported medication use. The majority of studies (64.2%) either excluded patients with co-morbid MDD or adjusted for this in analyses.

Concurrent physical illness is clearly an important determinant of inflammatory cytokine levels and this was accounted for by the majority of included studies by either excluding participants with co-morbidities (5 studies, 35.7%) or adjusting for chronic physical illness in group comparisons (6 studies, 42.8%), though two studies specifically only included participants with co-morbid cardiovascular disease. Use of a pre-determined cut-off value for cytokine levels was employed by three studies (21.4%) to ensure that cases with acute infection were excluded from the sample.

Many inflammatory markers exhibit a diurnal pattern of expression and are affected by consumption of food, thus time of day of sampling and whether the sample was taken in a fasted state are important factors to consider in analysing relative levels of cytokines[31]. However, time of day of sampling was only recorded in a minority of studies (6 studies, 42.8%), and the same number of studies recorded whether fasted samples were taken.

The overall quality of studies included in the review varied significantly, with Newcastle-Ottawa scale scores ranging from 2 to 9 (see Table 3). The area in which most studies were inadequate was in reporting non-response rate and detailing recruitment methods (see Table 3). Lowest quality studies were abstracts or dissertations, and two studies lacked control groups as only GAD patients were sampled (see Table4).

C-reactive protein

CRP is a critical early pro-inflammatory surveillance molecule involved in the activation of the complement system and both innate and adaptive immune systems[32]. We identified nine studies that investigated the association between GAD and CRP, comprising a total of 11,486 participants (see Table 5).

Four studies, involving 578 GAD patients and 4046 controls, provided sufficient information to conduct a meta-analysis of CRP levels in GAD[33–36] (see Figure 2). This was the only

For peer review only - http://bmjopen.bmj.com/site/about/guidelines.xhtml

inflammatory marker for which meta-analysis was possible. Meta-analysis showed significantly raised CRP in GAD compared to controls (SMD 0.38, 95% CI 0.06-0.69; Z=2.36, p=0.02). However, there was a large and statistically significant degree of heterogeneity between studies (Chi2=12.0; df=3; p=0.007; I^2 = 75%). Given the high heterogeneity and inclusion of less than 10 studies in the meta-analysis we did not have sufficient power to examine publication bias[37]. Two out of four studies were high quality, scoring 9 on the Newcastle-Ottawa scale, and examined large sample sizes[33,36] (see Table 4). However, in each of the four meta-analysed studies different assay methods were used, and sampling methods varied significantly (see Table 2). The lowest quality study to be included in the meta-analysis did not report mental health co-morbidities and recruited participants from an inpatient setting[34]. There was also a wide range in age of participants included in the four studies, with the largest study examining CRP levels in adolescents, that would likely contribute to high heterogeneity.

Five studies[33–36,38] (n=4669), one of which was conducted in 16 year olds[33] and two in participants with co-morbid heart disease[38], reported significantly higher CRP levels in participants with a diagnosis of GAD. The largest study[39] (n=5810) examining CRP in GAD examined CRP levels in children from baseline measurement aged 9-16 years to follow up aged 19-21.This was the only study to examine the longitudinal association between GAD and CRP, and found a bivariate association both cross-sectionally and over time between GAD and elevated CRP , however, this was accounted for by potential co-variates including BMI and medication use. The only study[40] to find an inverse correlation between CRP and GAD was conducted in non-smoking women from a study in Finland and did not specify the numbers of participants with a diagnosis of GAD or group differences.

No difference was found in a cohort study (n=821) that used a combined inflammatory index consisting of CRP, IL-6 and TNF-α in 93 patients with a diagnosis of GAD and controls with a history of cardiovascular disease (CVD)[41]. Subgroup analysis examining differences in individual inflammatory markers was not reported[41].

We found two studies[35,42] (n= 196) that examined the association of severity of GAD symptoms with CRP level. One found a significant positive correlation between CRP level and GAD-7 scores[35], and the other reporting CRP differences in 70 GAD patients with and without a diagnosis of alexithymia found a significant association between higher CRP and suicidal ideation[42].

Although the meta-analysis and the majority of included studies reported raised CRP in GAD, there was wide variation in reporting and adjustment for important potential moderators, including co-morbid MDD, use of medications, assay used and time of day of blood collection, all of which likely contributed to the high degree of heterogeneity between studies. Of the nine studies to analyse CRP, four (44.4%) did not exclude or adjust for medication use by participants[33,38,40,41]. Co-morbid MDD was not adjusted for in analysis by two studies[39,41], one of which was included in the meta-analysis[39]. Only three of the nine studies reported time of sample collection[35,36,42] or whether this was in a fasted state[36,41,42],

For peer review only - http://bmjopen.bmj.com/site/about/guidelines.xhtml

and though all studies utilised a similar assay method, different assay types were used in every study.

In summary, of the nine studies to have examined differences between GAD and controls, the majority reported raised CRP in GAD and meta-analysis found significantly raised CRP in GAD with a small effect size. However, there was wide variation in study methods including variable adjustment for mediators of inflammation such as co-morbid MDD, medication use and sampling methods. Only one study examined CRP in GAD longitudinally, reporting a bivariate association accounted for by health seeking behaviours[39].

Interleukins

Seven studies examined the association between interleukins and GAD (see Table 3). IL-6 is a mediator of T-cell and B-cell activation and induces acute phase proteins in hepatocytes, among other functions[32]. Pharmacological blockade of IL-6 action is used to treat several auto-immune conditions including RA, and raised IL-6 has been associated with a number of psychiatric conditions including depression, schizophrenia and PTSD[32,43]. We found IL-6 was the most frequently measured interleukin, with five studies (n=2066) examining changes in GAD patients compared to controls[35,36,41,44,45]. The largest study investigated differences between 454 participants with a diagnosis of GAD and 556 controls from The Netherlands Study of Depression and Anxiety (NESDA) cohort[36]. Though analysis was conducted on anxiety disorders as a whole, mean difference in IL-6 in people with GAD compared to controls obtained through direct communication with the author showed significantly higher levels in GAD. However, it is unclear whether these differences remain significant after adjustment for group differences and no associations were found between IL-6 and participants who had all types of anxiety disorder[36]. Two studies[35,45] (n=165), one of which used saliva samples[45], reported significantly higher IL-6 in medication naïve participants with a diagnosis of GAD compared to age and sex matched healthy controls.

No difference was found in a combined inflammatory index consisting of CRP, IL-6 and TNF-α in a study of 93 patients with GAD and co-morbid ischaemic heart disease[41]. One case-controlled study[35] of 48 Chinese outpatients presenting for the first time with a diagnosis of GAD and 48 age, sex and education matched controls accounted for all results for IL-1α, 5, 8, 12p70. This study found significantly higher levels of IL-1α, -8 and -12p70 in GAD patients, in addition to higher levels of IL-1α and IL-8 with increased severity of GAD (as measured by GAD-7 scale), but did not account for chronic physical co-morbidities during recruitment or in analysis. Both IL-1α and IL-8 have pro-inflammatory functions as chemo-attractants for leukocytes and haematopoiesis, and have been targeted for treatments in a number of auto-immune conditions[32]. However, there was no association between GAD and IL-5, which is thought to predominantly mediate myeloid cell activation, and is a target of treatment in asthma[32]. The same study[35] also examined IL-2, which has a major role in T-cell mediated autoimmune and inflammatory conditions[32]. Results showed significantly higher IL-2 in GAD patients, however, this conflicted with results from a smaller study (n=24) that found no significant difference between medication naïve GAD patients and controls, though

For peer review only - http://bmjopen.bmj.com/site/about/guidelines.xhtml

few details of the characteristics of participants, sampling or analysis were reported in this abstract[46].

One study that measured IL-1 using sputum analysis found significantly higher levels in GAD compared to controls in 69 participants recruited from the same Chinese hospital[45]. Though IL-1 is pro-inflammatory, there are differences in function dependent on the class of IL-1 protein measured which was not reported in this study[45].

IL-4 has several pro-inflammatory functions including immunoglobulin-E (Ig-E) class switching, expression of MHC class II and acts as a survival factor for T and B cells[32]. The only study to measure IL-4, found no differences between 54 GAD patients recruited from community mental health teams and primary care after controlling for age, sex, BMI, smoking, alcohol consumption and co-morbid depression[47]. This study[47] also investigated IL-10, which was the only cytokine with an anti-inflammatory function to be measured and is involved in immunosuppression of T cell subsets and B cell immunoglobulin production. This found significantly lower levels of IL-10 (OR 0.35 $p = 0.003$) in GAD patients. However, this opposed findings from a smaller study (n=24) which reported significantly higher levels of IL-10 in GAD patients compared to controls, though it was not reported whether this association remained significant after controlling for group differences[46].

In summary, IL-6 was the most commonly measured interleukin raised in GAD compared to controls in the majority of studies, however, no study examined the longitudinal association with GAD. Other interleukins were examined by relatively few studies that examined small numbers cross-sectionally with mixed findings.

IFN-γ

IFN-γ has anti-viral roles including promoting cytotoxic activity, MHC class I and II upregulation, NK cell activation, and is a treatment target in inflammatory conditions such as Crohn's disease[32]. Three studies investigated IFN-γ levels in GAD (n= 330)[35,40,47]. The largest study (n=118) found higher IFN-γ in GAD patients from the UK that remained significant after adjustment for age, gender, BMI, smoking, alcohol and co-morbid depression, but did not adjust for anxiolytic medication use in analysis[47]. This finding was supported by a study of 96 participants which reported higher IFN-γ levels in GAD and a significant positive correlation between anxiety severity and IFN-γ[35]. Conflicting findings were reported by a Finnish study of 116 participants, which found significantly lower IFN-γ in GAD patients. However, the number of participants with a diagnosis of GAD, differences between groups and adjustment for potential confounders were not reported[40]. In summary, only a few small cross-sectional studies have examined differences in IFN-γ between GAD and control groups, and their findings were mixed.

TNF-α

TNF-α has a wide array of roles in host defence, including initiating a strong acute inflammatory response but limiting duration of inflammatory activation, and is the target of blocking monoclonal antibodies in the treatment of a wide array of autoimmune conditions

For peer review only - http://bmjopen.bmj.com/site/about/guidelines.xhtml

including Crohn's disease and RA[32]. Six studies (n=2300) investigated TNF-α in GAD, with mixed findings. Three studies (n=303) found TNF-α significantly raised in GAD patients compared to controls[40,45,47]. However, the largest study to measure TNF-α (n=1010) found no difference between participants with GAD and controls, and no correlation between TNF-α and anxiety symptoms[36]. This finding was supported by a study of 93 patients with GAD and co-morbid ischaemic heart disease using a combined inflammatory index of CRP, IL-6 and TNF-α which reported no difference compared to controls[41]. In summary, though the majority of studies to measure differences in TNF-α between GAD and controls reported significantly raised levels, these comprised small cross-sectional studies and the largest study reported no difference.

Other cytokines

One study compared levels of the pro-inflammatory cytokines CCL-5, MCP-1 and SDF-1 in 120 medication naïve physically well patients with a diagnosis of GAD and co-morbid personality disorder to 40 controls[48]. Significantly higher levels of MCP-1 and SDF-1 were reported in both men and women, and higher CCL-5 in men but not women with a diagnosis of GAD compared to controls[48].

Discussion

To our knowledge, this is the first systematic review and meta-analysis focusing on inflammatory cytokines in GAD. Using a range of databases we identified 14 studies, comprising 1188 participants with GAD and which measured 16 cytokines. We found significantly raised levels of CRP, IFN-γ and TNF-α in people with GAD compared to controls which were findings replicated in two or more studies. A further 10 pro-inflammatory cytokines were reported to be significantly raised in GAD in at least one study, however, 6/14 studies found no difference in at least one cytokine.

Despite substantial efforts to acquire data by contacting authors, it was only possible to conduct a meta-analysis of CRP. This identified significantly higher levels in GAD compared to controls with a small effect size (SMD: 0.38), though there was evidence of significant heterogeneity across studies ($I^2 = 75\%$). This effect size in CRP is greater than has been reported in other anxiety disorders (PTSD: SMD = -0.14)[24] or MDD (SMD= 0.14)[6], and is similar to that reported in schizophrenia (SMD= 0.45)[49]. However, the effect size of our meta-analysis was driven by findings in poorer quality studies with small sample sizes. The two higher quality, larger studies reported a smaller effect size and no significant difference between groups respectively. As a result, further high quality studies are required to confirm our findings of raised CRP in GAD.
Though we were only able to meta-analyse CRP, meta-analyses of different cytokines in other anxiety disorders have been conducted with larger effect sizes. A meta-analysis of inflammatory markers in PTSD identified 20 studies which reported increased interleukin 6 (IL-6), interleukin 1β (IL-1β), tumour necrosis factor-α (TNFα), and interferon γ (IFN γ) levels with effect sizes ranging from small (IFN γ: SMD 0.49) to large 1.42 (IL-1β: SMD

For peer review only - http://bmjopen.bmj.com/site/about/guidelines.xhtml

1.42)[24]. However, a systematic review and meta-analysis of pro-inflammatory cytokines in OCD identified 12 studies, and concluded that there was a significant reduction in IL-1β with moderate effect size (SMD: -0.60, $p<0.001$), and only IL-6 levels were significantly increased after subgroup analysis in medication-free adults with OCD[7]. It is unclear whether this profile of inflammatory marker changes would follow a similar pattern in GAD if future studies enabled further meta-analysis.

In light of the high heterogeneity among studies, low participant numbers and inconsistent reporting and adjustment for known confounding factors such as BMI, smoking, medication use and co-morbidities our findings should be interpreted with caution. It was not possible to analyse the cause of the degree of heterogeneity due to the paucity of studies. Other known mediators of inflammation[24] such as physical activity, raised blood pressure and genetics were not accounted for. Furthermore, reporting of GAD severity and duration of symptoms was generally poor, preventing detailed analysis of whether inflammatory markers predicted outcomes and quality of life. We also found limitations in inclusion of specific demographics of participants with GAD. For example, despite GAD in older adults being prevalent and often treatment resistant[50,51,52], only two studies included participants over the age of 65, both of which only included patients with co-morbid ischaemic heart disease.

We are beginning to understand the interplay between cytokines, the immune system and mental health[1,53]. At a molecular level we are aware that pro-inflammatory cytokines, including IFN, IL-1β and TNF, can reduce the availability of monoamines by inducing expression of pre-synaptic reuptake pumps and inhibiting enzymes involved in monoamine synthesis[54], linking the monoamine theory of anxiety with inflammatory mechanisms. There is also a growing understanding of the relationship between systemic inflammation and the central nervous system (CNS)[1,55]. Microglial activation has been shown to be mediated by peripheral cytokines, and increased activation has been found in post-mortem studies of patients with MDD and schizophrenia[1]. No study we identified correlated inflammatory marker changes with in vivo microglial activation imaging in GAD and to our knowledge no research on post-mortem microglial changes in GAD has been conducted. Increased neuronal activity has also been shown to induce inflammatory and vascular changes in the brain, suggesting that psychological stress can not only be induced by inflammation but perpetuate chronic low grade inflammation seen in other vascular and neurodegenerative disorders[55]. Understanding interactions between the CNS and immune system, and identifying biomarkers of GAD offers potential for novel therapeutic approaches. The revolution of development of monoclonal-antibody therapies for inflammatory disorders[56] raises the possibility of repurposing these medications for trials in treatment resistant GAD if specific and consistent profiles of inflammatory biomarkers are identified.

However, it remains unclear as to whether inflammation plays a causal role in GAD[43,54]. For example, although IL-6 is a successful target for treatment in a number of auto-immune conditions and raised IL-6 is implicated in several psychiatric disorders, it also acts to reduce other pro-inflammatory cytokines such as TNF via negative feedback and is induced by physical exercise, hyperthermia, fasting, sleep deprivation and sunlight exposure without

For peer review only - http://bmjopen.bmj.com/site/about/guidelines.xhtml

activation other pro-inflammatory cytokines[43]. This raises the question as to whether inflammation in GAD is a consequence rather than cause of symptoms. This will only be answered by large prospective longitudinal studies, better characterising the relationship between inflammation and GAD. However, remarkably our review identified only one longitudinal study of inflammation in GAD patients that examined a cohort of adolescents until the age of 21 and only investigated CRP.

Recent studies using Mendelian randomisation in depression have suggested that cytokines such as IL-6 are causal risk factors for depression[57], and trials of immunotherapy in psychosis are already underway[58]. Our study suggests that GAD is an important candidate for future similar future studies exploring causality of inflammation and potentially novel drug trials.

Conclusion

There is some preliminary evidence to suggest a raised inflammatory response in GAD, although it is unclear whether inflammatory cytokines play a role in aetiology. GAD remains a poorly studied area of psychiatric neuroinflammatory research compared to other mental illnesses such as MDD and schizophrenia. While we are a long way from using inflammatory cytokines as a biomarker or treatment target in GAD, current findings reflect inflammatory changes seen in other mental illnesses and highlight the importance of ongoing investigation of the role inflammation plays in the development and course of GAD. Further, methodologically consistent, prospective, longitudinal studies examining the mechanisms and relationship between inflammation and GAD, while accounting for known mediators of cytokine production, are required.

Acknowledgements/ Funding Sources

HC and EA are NIHR Academic Clinical Fellows (ACF). This research was supported by the NIHR Biomedical Research Centre at University College London/University College Hospital London.

Declaration of interests

All authors declare no competing interests.

Contributors

RH, RG and HC were involved in initial design of the research. EA and HC performed data extraction of included studies and quality analysis. HC wrote the initial draft of the manuscript and did the statistical analysis with supervision from RG and RH. RH, RG and HC participated in the critical revision of the Article and all authors approved the final Article.

Data sharing

All data is available on request from the corresponding author.

For peer review only - http://bmjopen.bmj.com/site/about/guidelines.xhtml

For peer review only

Figure legends:

Figure 1. Flow of studies in the systematic review and meta-analysis.

Figure 2. Random effects meta-analysis of CRP levels in GAD vs controls.

For peer review only

Table 1. Study & clinical characteristics

Study	Country	Study type	Inflammatory markers	N GAD	N Control	Standardised diagnostic assessment/criteria	Anxiety measure	Age (years, SD) GAD	Age (years, SD) Control	% Female GAD	% Female Control	BMI GAD	BMI Control	Current smoking	Medicated	Physical health co-morbidities	Mental health co-morbidities
**Bankier et al. 2008**	US	Case-control	CRP	15	30	SCID, DSM IV	nr	nr	67.6 (12.7)	nr	33%	nr	nr	GAD: nr. Controls: 10%	nr	All participants had CVD. Excluded other conditions.	Excluded.
**Copeland et al. 2012**	US	Cohort, prospective	CRP	146	5664	Child and adolescent psychiatric assessment <16, Young adult psychiatric assessment >16. DSM IV criteria.	Total number of anxiety symptoms (range: 0-6)	14.21 All subjects (Odds or means ratio 1.12 (1.03-1.22) with GAD dx)		48.7% (Odds or means ratio 2.02 (1.04-3.92) with GAD dx)		22.37 (5.62) (Odds or means ratio 1.08 (1.05-1.11) with GAD dx)		Total sample: 13.5% (Odds/means ratio with GAD: 2.86)	30.2% 'use medication' (Odds/means ratio with GAD: 2.00)	34.7% had 'recent health ailments'.	Total sample: 39.9% co-morbid MDD.
**De Berardis et al. 2017**	Canada	Cross-sectional	CRP	70	no control	SCID, DSM IV	HAM-A (score >20 for inclusion)	28.2 (5.3)	-	51.40 %	-	22.1 (1.67)	-	nr	Excluded	Excluded	Total sample: 44.3% of participants had alexithymia. Excluded other co-mordid mental illness
**Hoge et al. 2016**	US	RCT	TNF-α, IL-6	70	no control	SCID, DSM IV	nr	39.12	-	45.70 %	-	nr	-	nr	Excluded	Excluded	GAD: 14.3% co-morbid MDD. Excluded other co-mordid mental illness
**Hou et al. 2017**	UK	Case-control	IL-4, IL-10, TNF-α, IFN-γ	54	64	MINI, DSM-IV & ICD-10 criteria	HADS, GAD-7 (score >10 for inclusion) .	35.06 (14.45)	25.75 (8.87)	34%	50%	24.84 (5.70)	22.45 (3.27)	GAD: 22% Controls: 34%	GAD: 67% use 'anxiolytic' medication. Excluded	Excluded.	Excluded

For peer review only

For peer review only - http://bmjopen.bmj.com/site/about/guidelines.xhtml

															other medication use.		
**Khandaker et al. 2016**	UK	Cohort, prospective	CRP	26	3392	DAWBA. DSM-IV criteria	DAWBA	15.56 (0.24)	15.53 (0.31)	90%	52.30 %	22.55 (3.52)	21.40 (3.63)	nr	nr	nr	GAD: 30.77% co-morbid MDD. Excluded other co-mordid mental illness.
**Korkelia et al. 2010**	Finland	Cross-sectional	CRP, TNF-α, IFN-γ	116		MINI	nr	nr	nr	100%	100%	All 25.3 (5.0)	nr	Excluded	nr	nr	nr
**Nayek et al 2016**	India	Case-control	CRP	50	50	ICD-10	nr	37.96 (10.7)	37.00 (12.08)	54%	23%	nr	nr	Excluded	Excluded if using HRT or OCP. Other medications not reported.	Excluded	nr
**Oglodek et al. 2015**	Poland	Case-control	SDF-1, CCL-5, MCP-1	120	40	DSM-V	nr	41.4 (3.5)	40.8 (3.1)	50%	nr	nr	nr	nr	Excluded	Excluded	All participants had co-morbid personality disorder. Excluded other co-mordid mental illness.
**Tang et al. 2017**	China	Case-control	CRP, IL-1α,, IL-2, IL-5, IL-6, IL-8, IL-12p70, IFN-γ, GM-CSF	48	48	MINI, DSM-IV	GAD-7, SAI, TAI	40.75 (12.21)	39.56 (10.06)	58.33 %	64.17 %	22.56 (2.73)	22.69 (2.63)	GAD: 29% . Controls: 23%	Excluded	Excluded acute illness. Chronic co-morbidities nr.	Excluded
**Tofani et al. 2015**	Italy	Case-control	IL-2, IL-10	14	10	MINI, DSM-IV	GAD-7	nr	nr	nr	nr	nr	nr	nr	Excluded	nr	Excluded

For peer review only

**Vogelzangs et al 2013**	Holland	Cohort	CRP, IL-6, TNF-α	454	556	CIDI, DSM criteria	BAI	Total sample: 41.8 (13.1)		66.90%		25.6 (5.1)		Total sample: 38.2%	nr	Total sample: 6.2% CVD, 4.9% diabetes, mean of 0.4 other chronic diseases.	Total sample: 58.4% co-morbid MDD. Excluded other co-mordid mental illness.
**Yang et al. 2017**	China	Case-control	IL-1, IL-4, TNF-α	28	41	MINI, DSM IV	HAM-A	55.1 (6.9)	55.9 (5.6)	53.60 %	48.80 %	22.0 (4.4)	22.5 (3.7)	GAD: 35.7%. Control: 24.4%	Excluded	Additional group with co-morbid asthma. Excluded other co-morbidities.	Excluded
**Zahm 2016**	US	Cohort, prospective	CRP, IL-6, TNF-α	93	728	CDIS, DSM IV	nr	68 (9.6)		17%		nr	nr	nr	nr	All patients had history of CVD.	GAD: 60.0% co-morbid MDD, 86.2% had lifetime history MDD.

not reported (nr), not applicable (-), sd: standard deviation, C-reactive protein (CRP), interleukin (IL-), tumour necrosis factor-α (TNF-α) , interferon-γ (IFN-γ), stromal derived factor-1 (SDF-1), monocyte chemoattractant protein-1 (MCP-1), chemokine C-C motif ligand 5 (CCL-5), granulocyte-macrophage colony-stimulating factor (GM-CSF), Diagnostic and Statistical Manual of Mental Disorders (DSM), International classification of diseases (ICD), Computerised diagnostic interview schedule (CDIS),Composite Interview Diagnostic Instrument (CIDI), Mini-International Neuropsychiatric Interview (MINI), Structure clinical interview for DSM IV (SCID), Development and wellbeing assessment (DAWBA), Montgomery and Asberg Depression Rating Scale (MADRS), Hamilton Anxiety Rating Scale (HAM-A), Hospital Anxiety and Depression Scale (HADS), Generalised Anxiety Disorder Assessment (GAD-7), State-Anxiety Inventory (SAI), Test Anxiety Inventory (TAI), Beck Anxiety Inventory (BAI), Cardiovascular disease (CVD), Major depressive disorder (MDD), Socio-economic status (SES), Body mass index (BMI), Oral contraceptive pill (OCP), Hormone replacement therapy (HRT)

For peer review only

Table 2. Inflammatory marker sampling and analysis

Study	GAD	Control	Inflammatory markers	Nature of sample	Cross-sectional or longtiduinal	Time of day of sample	Fasted period before sample	Assay method	Assay sensitivity reported	Inflammatory marker cutoff used.	Confounding factors controlled for
**Bankier et al. 2008**	15	30	CRP	Blood	Cross-sectional	nr	nr	High sensitivity tubidometric immunoassay	Yes	CRP >3mg/l for significance	Age, sex, education, MDD, obesity, smoking history, type II diabetes mellitus, hypertension, hyperlipidaemia, other mental illness.
**Copeland et al. 2012**	146	5664	CRP	Whole blood spots	Longitudinal: Sampled aged 9-16, 19, and 21 years old.	nr	nr	Biotin-Streptavidin based Immunofluorometric system.	Yes	Excluded if >10 mg/l	Age, sex, race, SES, BMI, medication use, substance use, recent physical illness, chronic illness.
**De Berardis et al. 2017**	70	no control	CRP	Serum	Cross-sectional	7-8.30am	10 hour fast	Highly sensitive nephelometric assay	Yes.	No	Age, sex, BMI, MDD, physical illness, other mental illness, medication use
**Hoge et al. 2016**	70	no control	TNF-α, IL-6	Plasma	Longitudinal: sampled pre- and post-psychological intervention	1-4.30pm	nr	nr	No	No	Age, sex, ethnicity, MDD, medication use, physical illness, other mental illness
**Hou et al. 2017**	54	64	IL-4, IL-10, TNF-α, IFN-γ	Serum	Cross-sectional	9-10am	nr	Multiplex ultra-sensitive immunoassay	Yes	No	Age, sex, BMI, smoking, alcohol consumption, MDD, physical illness, other mental illness
**Khandaker et al. 2016**	26	3392	CRP	Serum	Cross-sectional	nr	'Overnight'	Automated particle-enhanced immunoturbidimetric assay	No	Excluded if >10 mg/l	Age, sex, parental SES, ethnicity, maternal age at delivery, concurrent infection, family history of inflammatory disease, MDD.
**Korkelia et al. 2010**	116		CRP, TNF-α, IFN-γ	Blood	Cross-sectional	nr	nr	nr	No	No	BMI

For peer review only

**Nayek et al 2016**	50	50	CRP	Serum	Cross-sectional	nr	nr	Particle enhanced turbidimetric immunoassay technique	No	Excluded if 'raised ESR'	Age, sex, SES, religion, marital status, locality, BMI >30, physical illness
**Oglodek et al. 2015**	120	40	SDF-1, CCL-5, MCP-1	Plasma	Cross-sectional	7-9am	Fasted, duration nr.	ELISA	Yes	No	Sex, other mental illness, physical illness, substance misuse, smoking status, medication use.
**Tang et al. 2017**	48	48	CRP, IL-1α,, IL-2, IL-5, IL-6, IL-8, IL-12p70, IFN-γ, GM-CSF	Serum	Cross-sectional	9-10am	nr	ELISA	No	No	Age, sex, education, BMI, smoking status, alcohol consumption, acute physical illness, other mental illness, medication use.
**Tofani et al. 2015**	14	10	IL-2, IL-10	Plasma	Cross-sectional	nr	nr	Immunoenzymatic assay	No	No	Medication use.
**Vogelzangs et al 2013**	454	556	CRP, IL-6, TNF-α	Plasma	Cross-sectional	8-9am	'Overnight'	ELISA	Yes	No	Age, sex, education, smoking status, alcohol intake, physical activity, BMI, physical illness, medication use, MDD, other mental illness
**Yang et al. 2017**	28	41	IL-1, IL-4, IL-6, TNF-α	Saliva	Cross-sectional	nr	'Overnight'	ELISA	Yes	No	Age, sex, smoking status, BMI, medication use, physical illness, other mental illness
**Zahm 2016**	93	728	CRP, IL-6, TNF-α	Serum	Cross-sectional	No. (fasting, duration nr)	Fasted, duration nr.	ELISA	Yes	No	Age, sex, SES, BMI, illicit substance use, alcohol use, smoking status, physical activity, physical illness

nr (not reported), C-reactive protein (CRP), interleukin (IL-), tumour necrosis factor-α (TNF-α) , interferon-γ (IFN-γ), stromal derived factor-1 (SDF-1), monocyte chemoattractant protein-1 (MCP-1), chemokine C-C motif ligand 5 (CCL-5), granulocyte-macrophage colony-stimulating factor (GM-CSF), enzyme linked-immuno-sorbent assay (ELISA), erythrocyte sedimentation rate (ESR), mg (milligrams), l (litre), socioeconomic status (SES), body mass index (BMI), major depressive disorder (MDD)

For peer review only

Table 3. Summary inflammatory marker findings in GAD

Study	*N* Controls	n with GAD	Finding
			**C-reactive protein (CRP)**
Bankier et al. 2008	30	15	↑ in GAD with co-morbid CVD compared to controls using a dichotomous outcome of CRP cut off score (CRP >3mg/l).
Copeland et al. 2012	5664	146	Longitudinal study in adolescents: ↑ bivariate association both cross-sectionally & over time between GAD & elevated CRP, but accounted for by medication use & BMI.
De Berardis et al. 2017	no control	70	↑ in GAD patients with co-morbid alexithymia and with increased suicidal ideation, no control group.
Khandaker et al. 2016	3392	26	↑ in 16 year olds with GAD compared to controls, remained ↑ after adjusting for co-variates.
Korkelia et al. 2010	116		↓ in non-smoking women with diagnosis of GAD compared to controls, however control group not described.
Nayek et al. 2016	50	50	↑ in GAD patients compared to controls.
Tang et al. 2017	48	48	↑ in GAD patients compared to controls and ↑ with increased severity of GAD.
Vogelzangs et al 2013	556	454	↑ in GAD patients compared to controls in unadjusted data obtained from author.
Zahm 2016	728	93	↔ between those with & without a current GAD diagnosis (p = 0.28) or with & without a lifetime GAD diagnosis, using a combined inflammatory index of CRP, IL-6 & TNF-α measurements.
			**Interleukin 1 (IL-1)**
Yang et al. 2017	41	28	↑ sputum IL-1 in patients aged 50-60 years old with GAD compared to controls.
			**Interleukin 1α (IL-1α)**
Tang et al. 2017	48	48	↑ IL-1α in GAD patients compared to controls and ↑ with increased severity of GAD.
			**Interleukin 2 (IL-2)**
Tang et al. 2017	48	48	↑ in GAD patients compared to controls (p <0.001) but ↔ with severity of GAD.
Tofani et al. 2015	10	14	↔ in GAD patients compared to controls.
			**Interleukin 4 (IL-4)**
Hou et al. 2017	64	54	↔ in GAD patients compared to controls.
			**Interleukin 5 (IL-5)**
Tang et al. 2017	48	48	↔ in GAD patients compared to controls, or association with severity of GAD.
			**Interleukin 6 (IL-6)**
Hoge et al. 2016	-	70	No control group: RCT of psychological intervention in GAD
Tang et al. 2017	48	48	↑ in GAD patients compared to controls & ↑ with increased severity of GAD.
Vogelzangs et al 2013	556	454	↑ in GAD patients compared to controls in unadjusted data obtained from author, but ↔ between IL-6 & GAD compared to other anxiety disorders.
Yang et al. 2017	41	28	↑ sputum in GAD patients aged 50-60 years old compared to controls.
Zahm 2016	728	93	↔ between those with &without a current GAD diagnosis or with & without a lifetime GAD diagnosis, using a combined inflammatory index of CRP, IL-6 & TNF-α measurements.
			**Interleukin 8 (IL-8)**
Tang et al. 2017	48	48	↑ in GAD patients compared to controls and ↑ with increased severity of GAD.
			**Interleukin 10 (IL-10)**
Hou et al. 2017	64	54	↓ in GAD patients compared to controls, which remained ↓ after adjustment for co-variates.
Tofani et al. 2015	10	14	↑ in GAD compared to controls.
			**Interleukin 12p70 (IL-12p70)**
Tang et al. 2017	48	48	↑ in GAD patients compared to controls but ↔ with severity of GAD.
			**Interferon gamma (IFN-γ)**
Hou et al. 2017	64	54	↑ in GAD patients compared to controls which remained ↑ after adjustment for co-variates.
Korkelia et al. 2010	116		↓ in non-smoking women with diagnosis of GAD compared to controls, however control group not described.
Tang et al. 2017	48	48	↑ in GAD patients compared to controls and ↑ with increased severity of GAD.
			**Tumour necrosis factor-alpha (TNF-α)**
Hoge et al. 2016	-	70	No control group: RCT of psychological intervention in GAD

For peer review only - http://bmjopen.bmj.com/site/about/guidelines.xhtml

Hou et al. 2017	64	54	↑ in GAD patients compared to controls which remained ↑ after adjustment co-variates.
Korkelia et al. 2010	116		↑ in non-smoking women with a diagnosis of GAD compared to controls, though control group was not described.
Vogelzangs et al 2013	556	454	↔ in GAD patients compared to controls, & ↔ between TNF-α & GAD compared to other anxiety disorders.
Yang et al. 2017	41	28	↑ sputum TNF-α in GAD patients aged 50-60 years old compared to controls.
Zahm 2016	728	93	↔ between those with & without a current GAD diagnosis or with & without a lifetime GAD diagnosis, using a combined inflammatory index of CRP, IL-6 & TNF-α measurements.
			**Chemokine C-C motif ligand 5 (CCL-5) / Regulated on activation, normal T cell expressed and secreted (RANTES)**
Oglodek et al. 2015	40	120	↑ in males with GAD & comorbid personality disorder compared to controls
			**Monocyte chemoattractant protein-1 (MCP-1)**
Oglodek et al. 2015	40	120	↑ in GAD & comorbid personality disorder compared to controls
			**Stromal derived factor-1 (SDF-1)**
Oglodek et al. 2015	40	120	↑ in GAD & comorbid personality disorder compared to controls
			**Granulocyte-macrophage colony-stimulating factor (GM-CSF)**
Tang et al. 2017	48	48	↑ in GAD patients compared to controls and ↑ with increased severity of GAD.

↑ = statistically significant increase in inflammatory marker in people with GAD compared to controls ($p < 0.05$), ↓ = statistically significant decrease in inflammatory marker in people with GAD compared to controls ($p < 0.05$), ↔ = no statistically significant difference in inflammatory marker in people with GAD compared to controls ($p > 0.05$). RCT = randomised controlled trial.

For peer review only

Table 4. Study quality assessment: Newcastle Ottawa scale

	Selection				Comparability	Exposure			
	Adequate case definition	Cases representative	Selection of Controls	Definition of Controls	Comparability of design & analysis	Ascertainment of exposure	Same method of ascertainment	Non-response rate	TOTAL stars:
**Bankier et al. 2008**	◊	-	◊	◊	◊◊	◊	◊	◊	***8***
**Copeland et al. 2012**	◊	◊	◊	◊	◊◊	◊	◊	-	***8***
**De Berardis et al. 2017**	◊	-	na	na	na	◊	na	-	***2***
**Hoge et al. 2016**	◊	-	na	na	na	◊	na	-	***2***
**Hou et al. 2017**	◊	◊	◊	◊	◊◊	◊	◊	-	***8***
**Khandaker et al. 2016**	◊	◊	◊	◊	◊◊	◊	◊	◊	***9***
**Korkelia et al. 2010**	◊	-	◊	-	◊	-	◊	-	***4***
**Nayek et al 2016**	◊	-	◊	-	◊◊	◊	◊	-	***6***
**Oglodek et al. 2015**	◊	-	◊	◊	◊◊	-	◊	-	***6***
**Tang et al. 2017**	◊	◊	◊	◊	◊◊	◊	◊	-	***8***
**Tofani et al. 2015**	◊	-	-	-	◊	◊	◊	-	***4***
**Vogelzangs et al 2013**	◊	◊	◊	◊	◊◊	◊	◊	◊	***9***
**Yang et al. 2017**	◊	◊	-	◊	◊◊	◊	◊	-	***7***
**Zahm 2016**	◊	-	-	-	◊◊	◊	◊	-	***5***

◊ = met criteria, - = did not meet criteria, na = not applicable

For peer review only - http://bmjopen.bmj.com/site/about/guidelines.xhtml

Table 5. Additional critical appraisal

	Type of publication	Unrepresentative recruitment methods	Unrepresentative demographics	Between group differences reported	Adjusted for between-group differences
**Bankier et al. 2008**	Paper	Yes. Recruited from cardiology clinic.	Yes. Older cohort due to cardiac co-morbidity required.	nr	nr
**Copeland et al. 2012**	Paper	No.	Yes. Aged 9-21 only.	Yes	Yes
**De Berardis et al. 2017**	Paper	No	No (aged 18-45)	No control	No control
**Hoge et al. 2016**	Paper	Yes recruited by advert as part of parent RCT	No (aged >18)	No control	No control
**Hou et al. 2017**	Paper	No	No (aged 18-65)	Yes	Yes
**Khandaker et al. 2016**	Paper	No	Yes, aged 16 years old only	Yes	Yes
**Korkelia et al. 2010**	Abstract	Yes, recruited from existing study in Finland.	Yes. Non-smoking women only.	No	Yes (BMI only)
**Nayek et al 2016**	Paper	Yes, inpatients only	No, (aged 18-65)	Yes	Yes
**Oglodek et al. 2015**	Paper	Yes, co-morbid personality disorder	No	Yes	No
**Tang et al. 2017**	Paper	No	No, (aged 18-60)	Yes	Yes
**Tofani et al. 2015**	Abstract	Recruitment method not stated	nr	nr	nr
**Vogelzangs et al 2013**	Paper	No	No (aged 18-65)	Yes	Yes
**Yang et al. 2017**	Paper	No	Yes, (aged 50-60).	Yes	Yes
**Zahm 2016**	Dissertation	Yes, cardiology patients only.	Yes, (aged >50)	Yes	Yes

For peer review only

References

1. Réus GZ, Fries GR, Stertz L, et al. The role of inflammation and microglial activation in the pathophysiology of psychiatric disorders. *Neuroscience*. 2015. doi:10.1016/j.neuroscience.2015.05.018
2. Brown AS, Vinogradov S, Kremen WS, et al. Prenatal exposure to maternal infection and executive dysfunction in adult schizophrenia. *Am J Psychiatry*. 2009. doi:10.1176/appi.ajp.2008.08010089
3. Benros ME, Waltoft BL, Nordentoft M, et al. Autoimmune diseases and severe infections as risk factors for mood disorders: A nationwide study. *JAMA Psychiatry*. 2013. doi:10.1001/jamapsychiatry.2013.1111
4. O'dushlaine C, Rossin L, Lee PH, et al. Psychiatric genome-wide association study analyses implicate neuronal, immune and histone pathways. *Nat Neurosci*. 2015. doi:10.1038/nn.3922
5. Khandaker GM, Cousins L, Deakin J, Lennox BR, Yolken R, Jones PB. Inflammation and immunity in schizophrenia: Implications for pathophysiology and treatment. *The Lancet Psychiatry*. 2015. doi:10.1016/S2215-0366(14)00122-9
6. Howren MB, Lamkin DM, Suls J. Associations of depression with c-reactive protein, IL-1, and IL-6: A meta-analysis. *Psychosom Med*. 2009. doi:10.1097/PSY.0b013e3181907c1b
7. Gray SM, Bloch MH. Systematic review of proinflammatory cytokines in obsessive-compulsive disorder. *Curr Psychiatry Rep*. 2012. doi:10.1007/s11920-012-0272-0
8. Munkholm K, Vinberg M, Vedel Kessing L. Cytokines in bipolar disorder: A systematic review and meta-analysis. *J Affect Disord*. 2013. doi:10.1016/j.jad.2012.06.010
9. Meyer U, Feldon J. Neural basis of psychosis-related behaviour in the infection model of schizophrenia. *Behav Brain Res*. 2009. doi:10.1016/j.bbr.2008.12.022
10. Goldsmith DR, Rapaport MH, Miller BJ. A meta-analysis of blood cytokine network alterations in psychiatric patients: Comparisons between schizophrenia, bipolar disorder and depression. In: *Molecular Psychiatry*. ; 2016. doi:10.1038/mp.2016.3
11. Rao JS, Harry GJ, Rapoport SI, Kim HW. Increased excitotoxicity and neuroinflammatory markers in postmortem frontal cortex from bipolar disorder patients. *Mol Psychiatry*. 2010. doi:10.1038/mp.2009.47
12. Khairova RA, Machado-Vieira R, Du J, Manji HK. A potential role for pro-inflammatory cytokines in regulating synaptic plasticity in major depressive disorder. *Int J Neuropsychopharmacol*. 2009. doi:10.1017/S1461145709009924
13. Kubera M, Obuchowicz E, Goehler L, Brzeszcz J, Maes M. In animal models, psychosocial stress-induced (neuro)inflammation, apoptosis and reduced neurogenesis are associated to the onset of depression. *Prog Neuro-Psychopharmacology Biol Psychiatry*. 2011. doi:10.1016/j.pnpbp.2010.08.026
14. Furtado M, Katzman MA. Neuroinflammatory pathways in anxiety, posttraumatic stress, and obsessive compulsive disorders. *Psychiatry Res*. 2015. doi:10.1016/j.psychres.2015.05.036
15. Andlin-Sobocki P, Wittchen HU. Cost of anxiety disorders in Europe. *Eur J Neurol*. 2005. doi:10.1111/j.1468-1331.2005.01196.x
16. Baxter AJ, Scott KM, Vos T, Whiteford HA. Global prevalence of anxiety disorders: A systematic review and meta-regression. *Psychol Med*. 2013. doi:10.1017/S003329171200147X
17. Bystritsky A. Treatment-resistant anxiety disorders. *Mol Psychiatry*. 2006. doi:10.1038/sj.mp.4001852
18. Bruce SE, Yonkers KA, Otto MW, et al. Influence of psychiatric comorbidity on recovery and recurrence in generalized anxiety disorder, social phobia, and panic disorder: A 12-year prospective study. *Am J Psychiatry*. 2005. doi:10.1176/appi.ajp.162.6.1179
19. Baldwin D, Woods R, Lawson R, Taylor D. Efficacy of drug treatments for generalised anxiety disorder: Systematic review and meta-analysis. *BMJ*. 2011. doi:10.1136/bmj.d1199
20. Cuijpers P, Sijbrandij M, Koole S, Huibers M, Berking M, Andersson G. Psychological treatment of generalized anxiety disorder: A meta-analysis. *Clin Psychol Rev*. 2014. doi:10.1016/j.cpr.2014.01.002

For peer review only - http://bmjopen.bmj.com/site/about/guidelines.xhtml

21. Härter MC, Conway KP, Merikangas KR. Associations between anxiety disorders and physical illness. *Eur Arch Psychiatry Clin Neurosci*. 2003. doi:10.1007/s00406-003-0449-y
22. Roy-Byrne PP, Davidson KW, Kessler RC, et al. Anxiety disorders and comorbid medical illness. *Gen Hosp Psychiatry*. 2008. doi:10.1016/j.genhosppsych.2007.12.006
23. Uguz F, Akman C, Kucuksarac S, Tufekci O. Anti-tumor necrosis factor-α therapy is associated with less frequent mood and anxiety disorders in patients with rheumatoid arthritis. *Psychiatry Clin Neurosci*. 2009. doi:10.1111/j.1440-1819.2008.01905.x
24. Passos IC, Vasconcelos-Moreno MP, Costa LG, et al. Inflammatory markers in post-traumatic stress disorder: A systematic review, meta-analysis, and meta-regression. *The Lancet Psychiatry*. 2015. doi:10.1016/S2215-0366(15)00309-0
25. Michopoulos V, Powers A, Gillespie CF, Ressler KJ, Jovanovic T. Inflammation in Fear-and Anxiety-Based Disorders: PTSD, GAD, and beyond. *Neuropsychopharmacology*. 2017. doi:10.1038/npp.2016.146
26. Liberati A, Altman DG, Tetzlaff J, et al. The PRISMA statement for reporting systematic reviews and meta-analyses of studies that evaluate health care interventions: explanation and elaboration. In: *Journal of Clinical Epidemiology*. ; 2009. doi:10.1016/j.jclinepi.2009.06.006
27. Spitzer R, Williams J, Gibbon M, First M. Structured Clinical Interview for DSM-IV. In: *Encyclopedia of Behavioral Medicine*. ; 1994. doi:10.1007/978-1-4419-1005-9_66
28. Wells G, Shea B, O'Connell D, et al. *The Newcastle-Ottawa Scale (NOS) for Assessing the Quality of Nonrandomized Studies in Meta-Analyses.*; 2013. doi:10.2307/632432
29. Higgins JPT. Measuring inconsistency in meta-analyses. *BMJ*. 2003. doi:10.1136/bmj.327.7414.557
30. Kitahara CM, Trabert B, Katki HA, et al. Body mass index, physical activity, and serum markers of inflammation, immunity, and insulin resistance. *Cancer Epidemiol Biomarkers Prev*. 2014. doi:10.1158/1055-9965.EPI-14-0699-T
31. Petrovsky N, McNair P, Harrison LC. Diurnal rhythms of pro-inflammatory cytokines: Regulation by plasma cortisol and therapeutic implications. *Cytokine*. 1998. doi:10.1006/cyto.1997.0289
32. Akdis M, Aab A, Altunbulakli C, et al. Interleukins (from IL-1 to IL-38), interferons, transforming growth factor β, and TNF-α: Receptors, functions, and roles in diseases. *J Allergy Clin Immunol*. 2016. doi:10.1016/j.jaci.2016.06.033
33. Khandaker GM, Jones PB, Zammit S, Lewis G. Association between serum C-reactive protein and DSM-IV generalized anxiety disorder in adolescence: Findings from the ALSPAC cohort. *Neurobiol Stress*. 2016;4:55-61. doi:10.1016/j.ynstr.2016.02.003
34. Nayek S, Ghosh S. A Comparative Study Of Serum C-Reactive Protein In Patients With Generalised Anxiety Disorder And Depression. *Indian J Psychiatry*. 2017;59(6, 2):S217.
35. Tang Z, Ye G, Chen X, et al. Peripheral proinflammatory cytokines in Chinese patients with generalised anxiety disorder. *J Affect Disord*. 2018;225:593-598. doi:10.1016/j.jad.2017.08.082
36. Vogelzangs N, Beekman ATF, de Jonge P, Penninx BWJH. Anxiety disorders and inflammation in a large adult cohort. *Transl Psychiatry*. 2013;3:e249. doi:10.1038/tp.2013.27
37. Sterne JAC, Sutton AJ, Ioannidis JPA, et al. Recommendations for examining and interpreting funnel plot asymmetry in meta-analyses of randomised controlled trials. *BMJ*. 2011. doi:10.1136/bmj.d4002
38. Bankier B, Barajas J, Martinez-Rumayor A, Januzzi JL. Association between C-reactive protein and generalized anxiety disorder in stable coronary heart disease patients. *Eur Heart J*. 2008;29(18):2212-2217. doi:10.1093/eurheartj/ehn326
39. Copeland WE, Shanahan L, Worthman C, Angold A, Costello EJ. Generalized anxiety and C-reactive protein levels: A prospective, longitudinal analysis. *Psychol Med*. 2012;42(12):2641-2650. doi:10.1017/S0033291712000554
40. Korkeila J, Runsten S, Ollikainen S, Korkeila K. Generalized anxiety disorder and immunity markers in a stratified population sample. *Eur Psychiatry*. 2010;25. doi:10.1016/S0924-9338%2810%2971560-1
41. Zahm JL. Generalized anxiety disorder and inflammatory biomarkers in coronary heart disease: Sex-specific effects. *Diss Abstr Int Sect B Sci Eng*. 2018;78(12).
42. De Berardis D, Serroni N, Campanella D, et al. Alexithymia, suicide ideation, C-Reactive Protein, and serum lipid levels among outpatients with generalized anxiety disorder. *Arch Suicide Res*. 2017;21(1):100-112. doi:10.1080/13811118.2015.1004485

For peer review only - http://bmjopen.bmj.com/site/about/guidelines.xhtml

43. Raison CL, Knight JM, Pariante C. Interleukin (IL)-6: A good kid hanging out with bad friends (and why sauna is good for health). *Brain, Behavior, and Immunity*. 2018.
44. Hoge EA, Bui E, Palitz SA, et al. The effect of mindfulness meditation training on biological acute stress responses in generalized anxiety disorder. *Psychiatry Res*. 2017:No. doi:10.1016/j.psychres.2017.01.006
45. Yang CJ, Liu D, Du YJ, Xu ZS, Shi SX. The pro-inflammatory cytokines, salivary cortisol and alpha-amylase are associated with generalized anxiety disorder (GAD) in patients with asthma. *Neurosci Lett*. 2017;656:15-21. doi:10.1016/j.neulet.2017.07.021
46. Tofani T, Pallanti S, Di Cesare Mannelli L, Zanardelli M, Ghelardini C. An immunologic profile study in drug-naive generalized anxiety non depressed patients: A pilot study. *Eur Neuropsychopharmacol*. 2015;25.
47. Hou R, Garner M, Holmes C, et al. Peripheral inflammatory cytokines and immune balance in Generalised Anxiety Disorder: Case-controlled study. *Brain Behav Immun*. 2017;62:212-218. doi:10.1016/j.bbi.2017.01.021
48. Oglodek EA, Szota AM, Araszkiewicz A, Just MJ, Mos DM. The MCP-1, CCL-5 and SDF-1 chemokines as pro-inflammatory markers in generalized anxiety disorder and personality disorders. *Pharmacol Reports*. 2015;67(1):85-89. doi:10.1016/j.pharep.2014.08.006
49. Miller BJ, Culpepper N, Rapaport MH. C-reactive protein levels in schizophrenia: A review and meta-analysis. *Clin Schizophr Relat Psychoses*. 2014. doi:10.3371/CSRP.MICU.020813
50. Byers AL, Yaffe K, Covinsky KE, Friedman MB, Bruce ML. High occurrence of mood and anxiety disorders among older adults: The National Comorbidity Survey Replication. *Arch Gen Psychiatry*. 2010. doi:10.1001/archgenpsychiatry.2010.35
51. Wolitzky-Taylor KB, Castriotta N, Lenze EJ, Stanley MA, Craske MG. Anxiety disorders in older adults: A comprehensive review. *Depress Anxiety*. 2010. doi:10.1002/da.20653
52. Wetherell JL, Liu L, Patterson TL, et al. Acceptance and Commitment Therapy for Generalized Anxiety Disorder in Older Adults: A Preliminary Report. *Behav Ther*. 2011. doi:10.1016/j.beth.2010.07.002
53. Felger JC, Lotrich FE. Inflammatory cytokines in depression: Neurobiological mechanisms and therapeutic implications. *Neuroscience*. 2013. doi:10.1016/j.neuroscience.2013.04.060
54. Miller AH, Raison CL. The role of inflammation in depression: From evolutionary imperative to modern treatment target. *Nat Rev Immunol*. 2016. doi:10.1038/nri.2015.5
55. Xanthos DN, Sandkühler J. Neurogenic neuroinflammation: Inflammatory CNS reactions in response to neuronal activity. *Nat Rev Neurosci*. 2014. doi:10.1038/nrn3617
56. Reichert JM, Rosensweig CJ, Faden LB, Dewitz MC. Monoclonal antibody successes in the clinic. *Nat Biotechnol*. 2005. doi:10.1038/nbt0905-1073
57. Khandaker GM, Zuber V, Rees JM, et al. Shared mechanisms between coronary heart disease and depression: findings from a large UK general population-based cohort. *bioRxiv*. 2019. doi:10.1101/533828
58. Miller BJ, Buckley PF. The Case for Adjunctive Monoclonal Antibody Immunotherapy in Schizophrenia. *Psychiatr Clin North Am*. 2016. doi:10.1016/j.psc.2016.01.003

For peer review only

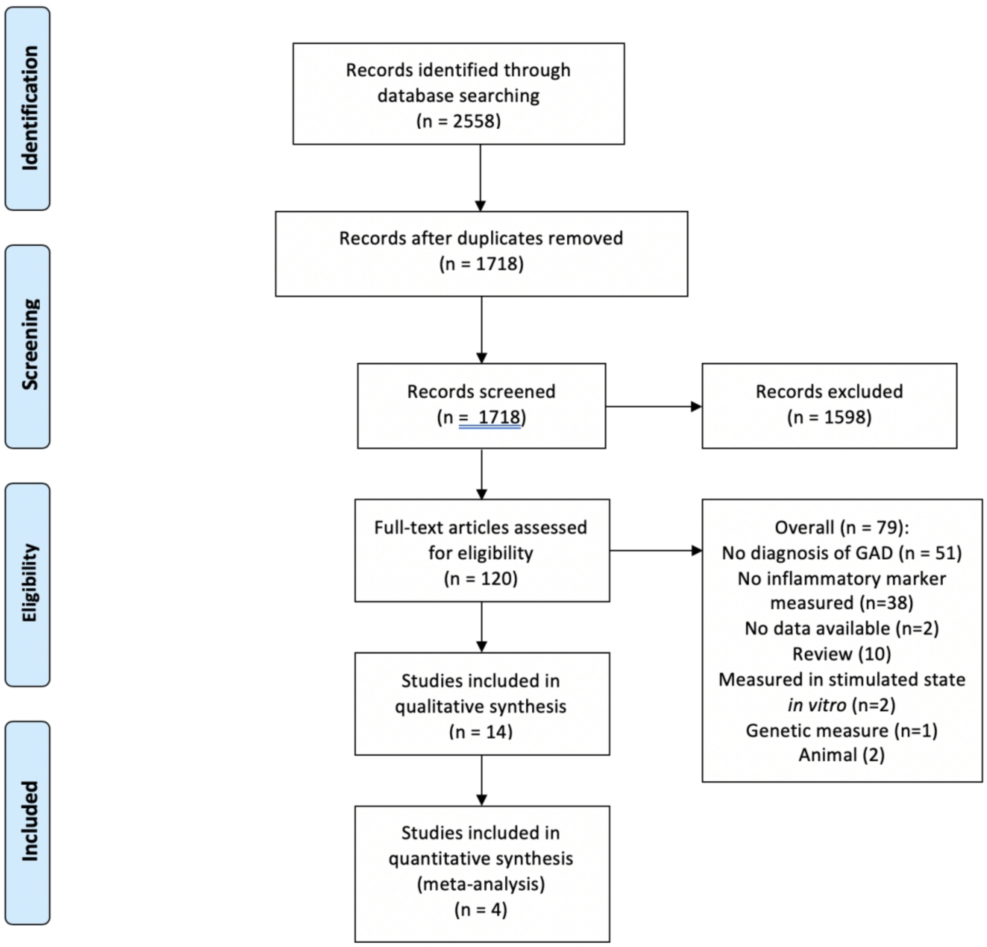

Figure 1. Flow of studies in the systematic review and meta-analysis

	GAD			Control				Std. Mean Difference
**Study or Subgroup**	**Mean**	**SD**	**Total**	**Mean**	**SD**	**Total**	**Weight**	**IV, Random, 95% CI**
Khandaker 2010	0.91	2.97	26	0.39	10.1	3392	22.9%	0.05 [-0.33, 0.44]
Nayek 2016	0.29	0.14	50	0.2	0.1	50	22.1%	0.73 [0.33, 1.14]
Tang 2017	1.19	0.8	48	0.68	0.7	48	21.9%	0.67 [0.26, 1.08]
Vogelzangs 2013	3.1	4.9	454	2.3	4.4	556	33.1%	0.17 [0.05, 0.30]
**Total (95% CI)**			**578**			**4046**	**100.0%**	**0.38 [0.06, 0.69]**

Heterogeneity: Tau² = 0.07; Chi² = 12.00, df = 3 (P = 0.007); I² = 75%
Test for overall effect: Z = 2.36 (P = 0.02)

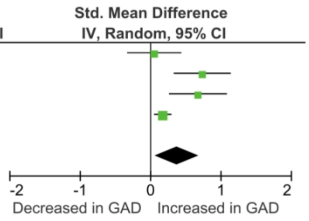

Figure 2. Forest plot of random effects meta-analysis of CRP levels in GAD vs controls.

MEDLINE SEARCH STRATEGY:
(“inflammat*” OR “cytokine” OR “interferon” OR “IFN” OR “interleukin” OR “translocator protein” OR “TSPO” OR “tumour necrosis factor” OR “tumor necrosis factor” OR “TNF” OR “IL-1” OR “IL-2” OR “IL-4” OR “IL-7” OR “IL-6” OR IL-8 OR IL-10 OR migroglia OR t-cell OR lymphocyte OR “C-reactive protein” OR “C reactive protein” OR CRP OR “acute phase protein” OR “fibrinogen”) AND (“generalised anxiety disorder” OR “generalized anxiety disorder” OR “GAD” OR “worry”).

For peer review only

PRISMA 2009 Checklist

Section/topic	#	Checklist item	Reported on page #
**TITLE**			
Title	1	Identify the report as a systematic review, meta-analysis, or both.	1
**ABSTRACT**			
Structured summary	2	Provide a structured summary including, as applicable: background; objectives; data sources; study eligibility criteria, participants, and interventions; study appraisal and synthesis methods; results; limitations; conclusions and implications of key findings; systematic review registration number.	2
**INTRODUCTION**			
Rationale	3	Describe the rationale for the review in the context of what is already known.	2-3
Objectives	4	Provide an explicit statement of questions being addressed with reference to participants, interventions, comparisons, outcomes, and study design (PICOS).	3-4
**METHODS**			
Protocol and registration	5	Indicate if a review protocol exists, if and where it can be accessed (e.g., Web address), and, if available, provide registration information including registration number.	3-4
Eligibility criteria	6	Specify study characteristics (e.g., PICOS, length of follow-up) and report characteristics (e.g., years considered, language, publication status) used as criteria for eligibility, giving rationale.	4
Information sources	7	Describe all information sources (e.g., databases with dates of coverage, contact with study authors to identify additional studies) in the search and date last searched.	4
Search	8	Present full electronic search strategy for at least one database, including any limits used, such that it could be repeated.	4
Study selection	9	State the process for selecting studies (i.e., screening, eligibility, included in systematic review, and, if applicable, included in the meta-analysis).	4
Data collection process	10	Describe method of data extraction from reports (e.g., piloted forms, independently, in duplicate) and any processes for obtaining and confirming data from investigators.	4
Data items	11	List and define all variables for which data were sought (e.g., PICOS, funding sources) and any assumptions and simplifications made.	4
Risk of bias in individual studies	12	Describe methods used for assessing risk of bias of individual studies (including specification of whether this was done at the study or outcome level), and how this information is to be used in any data synthesis.	5
Summary measures	13	State the principal summary measures (e.g., risk ratio, difference in means).	5
Synthesis of results	14	Describe the methods of handling data and combining results of studies, if done, including measures of consistency (e.g., I^2) for each meta-analysis.	5

For peer review only - http://bmjopen.bmj.com/site/about/guidelines.xhtml

PRISMA 2009 Checklist

Section/topic	#	Checklist item	Reported on page #
Risk of bias across studies	15	Specify any assessment of risk of bias that may affect the cumulative evidence (e.g., publication bias, selective reporting within studies).	4-5
Additional analyses	16	Describe methods of additional analyses (e.g., sensitivity or subgroup analyses, meta-regression), if done, indicating which were pre-specified.	5
**RESULTS**			
Study selection	17	Give numbers of studies screened, assessed for eligibility, and included in the review, with reasons for exclusions at each stage, ideally with a flow diagram.	5
Study characteristics	18	For each study, present characteristics for which data were extracted (e.g., study size, PICOS, follow-up period) and provide the citations.	5
Risk of bias within studies	19	Present data on risk of bias of each study and, if available, any outcome level assessment (see item 12).	5-6
Results of individual studies	20	For all outcomes considered (benefits or harms), present, for each study: (a) simple summary data for each intervention group (b) effect estimates and confidence intervals, ideally with a forest plot.	6
Synthesis of results	21	Present results of each meta-analysis done, including confidence intervals and measures of consistency.	6
Risk of bias across studies	22	Present results of any assessment of risk of bias across studies (see Item 15).	5-6
Additional analysis	23	Give results of additional analyses, if done (e.g., sensitivity or subgroup analyses, meta-regression [see Item 16]).	6
**DISCUSSION**			
Summary of evidence	24	Summarize the main findings including the strength of evidence for each main outcome; consider their relevance to key groups (e.g., healthcare providers, users, and policy makers).	6-11
Limitations	25	Discuss limitations at study and outcome level (e.g., risk of bias), and at review-level (e.g., incomplete retrieval of identified research, reporting bias).	6-11
Conclusions	26	Provide a general interpretation of the results in the context of other evidence, and implications for future research.	11-12
**FUNDING**			
Funding	27	Describe sources of funding for the systematic review and other support (e.g., supply of data); role of funders for the systematic review.	12

From: Moher D, Liberati A, Tetzlaff J, Altman DG, The PRISMA Group (2009). Preferred Reporting Items for Systematic Reviews and Meta-Analyses: The PRISMA Statement. PLoS Med 6(7): e1000097. doi:10.1371/journal.pmed1000097

For more information, visit: www.prisma-statement.org.

For peer review only - http://bmjopen.bmj.com/site/about/guidelines.xhtml
